# Combining HPLC-DAD-QTOF-MS and HPLC-SPE-NMR to Monitor In Vitro Vitetrifolin D Phase I and II Metabolism

**DOI:** 10.3390/metabo11080529

**Published:** 2021-08-09

**Authors:** Sonja Sturm, Christina Högner, Christoph Seger, Hermann Stuppner

**Affiliations:** Institute of Pharmacy/Pharmacognosy, CCB—Centrum of Chemistry and Biomedicine, University of Innsbruck, Innrain 80-82, A-6020 Innsbruck, Austria; hoegner.ch@gmail.com (C.H.); seger_lab@gmx.at (C.S.); Hermann.Stuppner@uibk.ac.at (H.S.)

**Keywords:** metabolism, liver microsomes, structure elucidation, HPLC-DAD-QTOF-MS, HPLC-SPE-NMR, HR-mass spectrometry, vitetrifolin D, *Vitex agnus-castus*

## Abstract

By combining HPLC-DAD-QTOF-MS and HPLC-SPE-NMR, the in vitro metabolism of vitetrifolin D, a pharmacologically active key molecule from *Vitex agnus-castus* in liver cell fractions, was investigated. Twenty-seven phase I and phase II metabolites were tentatively identified from the culture broth by HPLC-DAD-QTOF-MS. The subsequent HPLC-SPE-NMR analysis allowed for the unequivocal structural characterization of nine phase I metabolites. Since the preparative isolation of the metabolites was avoided, the substance input was much lower than in conventional strategies. The study did prove that the use of hyphenated instrumental analysis methodologies allows for the successful performance of in vitro metabolism studies, even if the availability of substances is very limited.

## 1. Introduction

The metabolism of xenobiotics is a basic evolutionary concept that lifeforms at any level of complexity use to successfully detoxify ingested organic matter. The molecular mechanisms involved are scientifically well investigated. This knowledge is the fundament of the pharmacokinetic characterization of new drugs in drug development. In such research, the transformation of the applied parent compound to one or more metabolites is analyzed in detail, mass balances are prepared, and the excretion pathways are described [1,2]. If, in mammalians, a xenobiotic can reach the bloodstream, the enzymes of the liver are mainly responsible for transforming these molecules. The general goal of the metabolism is to improve hydrophilicity to allow for excretion via the kidneys. In the first phase of the metabolism, functional groups are added (e.g., hydroxylation at single or double bonds) or existing functional groups are changed (e.g., demethylation of methoxy groups). In the second phase of the metabolism, conjugation with endogenous molecules with a hydrophilic character (e.g., sulfation, glucuronidation) occurs. All the involved enzymes show a relatively low, and often overlapping, substance specificity. Predicting the metabolic pathways is, therefore, difficult and the structural characterization of metabolites is widely accepted as inevitable in the deeper understanding of metabolic pathways [3,4]. Therefore, a particular challenge is the question of which of the substances found in the very complex metabolic mixture are actually metabolites of the added xenobiotic. This is particularly problematic if one cannot resort to starting compounds labeled with unstable isotopes such as ^14^C. These are traditionally used in pharmacological research as analytically highly selective tracing probes, but require a synthetic approach, which is often not easily achieved with natural substances [5,6,7].

Human medical metabolism studies of natural products are usually limited to the secondary metabolites, which are used as single-substance drugs, e.g., in cancer therapy, as anti-infectives or as immunosuppressants [8]. The metabolic fate of compounds from herbal medicines (“botanicals”) is not well covered by research [9,10], although its importance is understood [11,12,13,14]. The lack of data on, and lack of understanding of, the pharmacokinetic interaction of herbal drug constituents with the drugged organism is known as one of the major hurdles in the development of herbal medicines as fully accepted mainstream drugs/therapeutics. Although at least the in vitro, ex vivo, and in vivo bioactivity of extracts or purified compounds have been evaluated in detail for many well-established herbal medicines, the fate of the single secondary metabolites in uptake and metabolic transformation is not deeply understood. Hence, it is necessary for modern pharmacognosy to bridge the knowledge gap between chemical analysis/the metabolic dereplication of medicinal plant extracts and the pharmacodynamic effects/proven bioactivities of the applied remedies [15].

Modern high-resolution mass spectrometry (HR-MS) is one of the major technologies utilized in the analysis of unknown organic material. It is the only technology enabling the separation science researcher to deduce the elemental composition and, therefore, the elemental formula of a chromatographic feature (“chromatographic peak”), which—providing there is sufficient chromatographic resolution—represents a single molecule species. The 2D- or 3D-chemical structure of the molecule, however, can rarely be deduced from HR-MS data. By comparison, with databases or by the application of rulesets, such as isotope pattern analysis or data filtering by metabolism-associated nominal mass shifts [16], the data from HR-MS spectra can be filtered, such that the chromatographic features can be associated with sum formulae. If sum formula data are put into the context of compound/metabolite classes (e.g., the metabolites of a parent compound or lipids of a certain lipid class), the tentative assignment of a chromatographic peak as a molecule of interest is feasible [17,18].

However, as long as no comparison with a reference database or a reference standard can confirm the identity of a candidate molecule, the identification must continue to be designated as tentative. In research approaches dedicated to the metabolic fate of molecules that have never been investigated in such experiments, HR-MS does not usually allow for substance identification. Therefore, the HR-MS-based labeling of chromatographic features as putative metabolites of a parent substance must be combined with a technology whose main application is the structural characterization of unknown organic molecules in solution—NMR spectroscopy is such a method with well-understood possibilities and limitations [19]. Besides its unquestioned use in plant science for structure elucidation [20], it is a major cornerstone of the dereplication of mixtures, as encountered in metabolomics, metabolism studies, or bioactivity-guided fractionation efforts [21].

If, in such research approaches, putative biomarkers or putative active principles of a mixture must be assigned to a molecular entity, a structural characterization is inevitable. When phytoanalytical research involves working with very small sample quantities, the isolation of marker molecules is often not possible, for reasons of time and cost. In such cases, it is advisable to undertake identification with an online coupling of the analytical separation and the identifying NMR spectroscopy—the liquid chromatography–nuclear magnetic resonance (LC-NMR) hyphenation. NMR spectroscopy is an inherently insensitive detection method, limiting on-flow LC-NMR experiments to highly concentrated samples and ^1^H-NMR spectroscopy. To allow for longer experiment times or for heteronuclear shift correlation experiments, LC-NMR hyphenation has matured over the recent decades, such that chromatographic peaks of interest can be collected and stored after separation until measurement [22,23].

In the setup of the high-performance liquid chromatography–solid-phase extraction–nuclear magnetic resonance (HPLC-SPE-NMR) hyphenation, strictly online technology lipophilic SPE cartridges serve as storage devices between the LC separation domain and the NMR analysis domain (Scheme S1). Analytes are trapped after chromatographic separation onto the SPE phase by the aid of a hydrophilic post-LC-column added makeup flow. The absorbed analyte is dried with nitrogen to remove the chromatography solvents. Online transfer to the flow cell of the NMR probe head is facilitated by a deuterated NMR solvent (e.g., CD_3_CN, CD_3_OD), peak handling (e.g., transfer to NMR tubes) is strictly avoided. Due to the lipophilicity of the NMR solvent, sharp elution profiles can be realized from the SPE phase. This leads to a re-focusing of chromatographic peaks and a high analyte concentration in the flow cell. The liquid transfer to the NMR is done such that peak diffusion is minimized. The research approach presented in this publication combines the HPLC-DAD-QTOF-MS-based tentative identification of metabolites formed in an in vitro analyte metabolism experiment with the subsequent structural characterization of these metabolites utilizing a HPLC-SPE-NMR instrumentation setup.

**Scheme 1:**
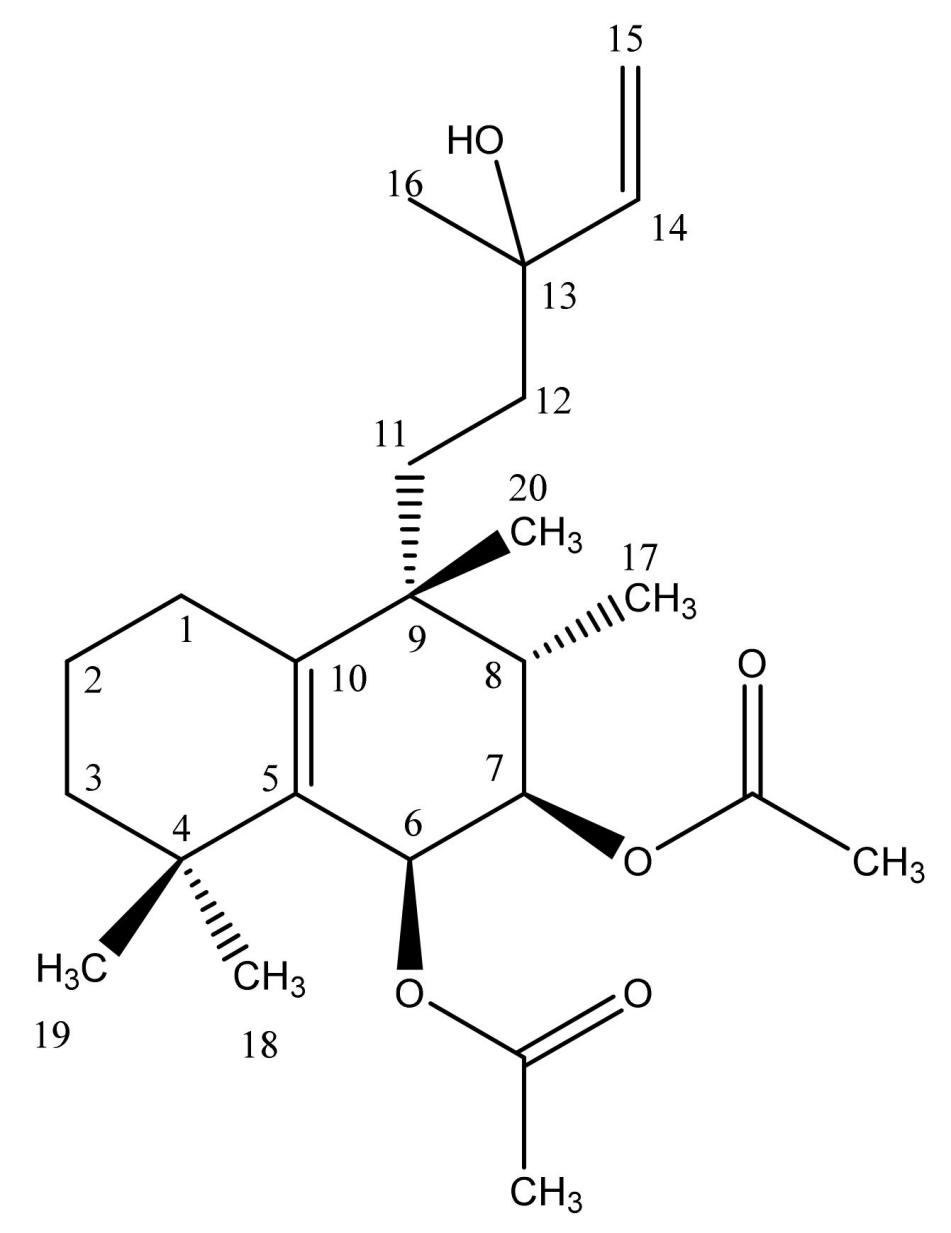
Molecular structure of the halimane diterpene vitetrifolin D (VD).

The model analyte chosen for this experiment was vitetrifolin D (VD) (Scheme 1), a diterpenoid secondary plant metabolite isolated from the herbal medicinal plant species *Vitex agnus-castus* L. (Lamiaceae). For the fruits of *V. agnus-castus*, which were for centuries in traditional use to treat gynecological problems, and specifically premenstrual complaints [24], different modes of action are discussed. Besides the well-studied interactions of flavones with ß-estrogenic receptors, the inhibition of dopamine and opioid receptors was mediated by extracts of different polarity. Diterpenoid-containing fractions and purified diterpenoids, including VD, showed dopamine D2 receptor activity inhibition. This, in turn, reduced the increased prolactin secretion associated with the female reproductive system diseases and triggered by dopamine [25,26,27]. These in vitro findings were substantiated by several clinical studies showing that premenstrual syndrome (PMS) is associated with a latent hyper-prolactinemia, which could be regulated to physiological values by taking preparations of *Vitex agnus-castus* extracts [24]. For these reasons, VD was selected as a relevant model substance for investigating the metabolism of *Vitex* terpenoids [28].

The biological system of commercially available standardized human liver microsomes (HLMs) was chosen for in vitro metabolite generation. Microsomes are vesicle-like artefacts reformed from the endoplasmic reticulum, in which proteins and lipids, as well as molecules for export, are synthesized, and in which biotransformation reactions of the monooxygenase enzyme systems occur [29,30]. Their use as a critical experimental model for the evaluation of drug metabolism in pharmacology and toxicology is well documented and standardized procedures are available [31,32].

## 2. Results

### 2.1. HPLC-DAD-QTOF-MS Analysis

VD is a halimane diterpene with two double bonds, two acetylated hydroxy functions at C-6 and C-7, and an unsubstituted hydroxy-function at C13 (Scheme 1). The mass spectrometric characterization of VD and its metabolites was performed using an HPLC-DAD-QTOF-MS instrumentation setup. Under the developed chromatographic conditions, VD was eluted at 28.77 min. Since more polar VD derivatives were expected in the in vitro experiments due to the inherent character of phase I and II metabolism, it was hypothesized that all metabolites would be eluted prior to the parent compound VD. Both electrospray ionization (positive ESI mode, +ESI) and atmospheric-pressure chemical ionization (positive APCI mode) were experimentally available as possible ionization techniques. In the design phase of the investigation, full-scan (MS) ESI and APCI spectra of VD, recorded under carefully optimized conditions, revealed that significant in-source fragmentation of the terpenoid compound reduced the applicability of APCI for the deduction of molecular masses from mass spectrometry data. Hence, this ionization technique was abandoned and all further experiments were performed under +ESI-based ion formation. High mass accuracy (<5 ppm) was achieved via calibration with sodium formate infused into the HPLC column effluent in a calibration time window at the beginning of each analysis. 

The +ESI MS spectrum of VD exhibited characteristic adduct ions at *m/z* 424.2819 ([M + H_2_O]^+^, error 0.1 ppm), 429.2597 ([M + Na]^+^, error 3.4 ppm), and 445.2339 ([M + K]^+^, error 2.7 ppm), confirming the VD molecular weight of 406.26 Da and the elemental composition of C_24_H_38_O_5_ (Table 1). Moreover, due to in-source fragmentations, the successive loss of the two acetoxy-groups at *m/z* 329.2475 ([M − OAc − H_2_O]^+^), 287.2369 ([M − 2OAc]^+^), and 269.2231 ([M − 2OAc − H_2_O]^+^) was observed (Scheme S2). MS/MS spectra recorded for each adduct ion over the course of the design phase of the investigation (data not shown) revealed a fragmentation pattern similar to the in-source fragmentation MS spectra. Diagnostic ions from halimane scaffold cleavages were not observed. Hence, MS spectra, optimized to molecular mass detection via [M + Na]^+^ and [M + K]^+^ adduct ions, were subsequently recorded for metabolite characterization.

Combining the recorded mass spectrometry data for VD proved that the chosen experimental conditions allowed for the deduction of molecular weight and elementary composition of VD without structural integrity loss of the analyte. In addition, in-source fragmentation of labile acetate C-O bonds did allow for a deeper, but limited, insight into the VD functional group substitution pattern. Neither the exact localization of the two acetate moieties, nor the detection of signals indicating the presence of the hydroxy functions of VD of the molecule were achievable. Nevertheless, since phase I and phase II metabolism only lead to changes in the substitution pattern, it was assumed that the experimental conditions for VD molecular mass detection and the sum formula calculation are also suitable for monitoring such metabolites.

Optimization of the in vitro VD incubation with pooled female human liver microsomes (HLMs) using three different VD concentrations (50 µM, 100 µM, 150 µM) and incubation times of up to 72 h revealed optimal results for 100 µM VD incubated for 24 h. Under these conditions, approximately 80% of VD was transformed and a stable phase I metabolite pattern was observable (Figure S1). Prolonged incubation or altered VD concentrations did not significantly improve the metabolite yield. The presence of residual VD in the reaction mixtures served as an internal standard for spectroscopic data interpretation. Similar observations were made in the optimization phase of the S9 cell incubation experiments. Consequently, identical reaction conditions were chosen for the phase II metabolite generation experiments. 

### 2.2. HPLC-DAD-QTOF-MS-Based Metabolite Characterization

As expected, in vitro metabolism resulted in a multitude of +ESI mass spectrometry detectable chromatographic features eluting in the retention time window of 11–22 min. Through application of the metabolite identification algorithm, provided by the Metabolite Tool software package, a tentative assignment of these features was undertaken. Based on the VD structure (Scheme 1) and the knowledge of phase I and phase II bioreactions [18], a tailored sum formula and molecular mass forecast list was generated with the Metabolite Predict algorithm. This forecast list served as a data filter, applied to the experimental HR-MS data using the Metabolite Detect algorithm. The output from the filtering procedure was a chromatographic features and mass spectra list of putative metabolite candidates.

**Figure 1:**
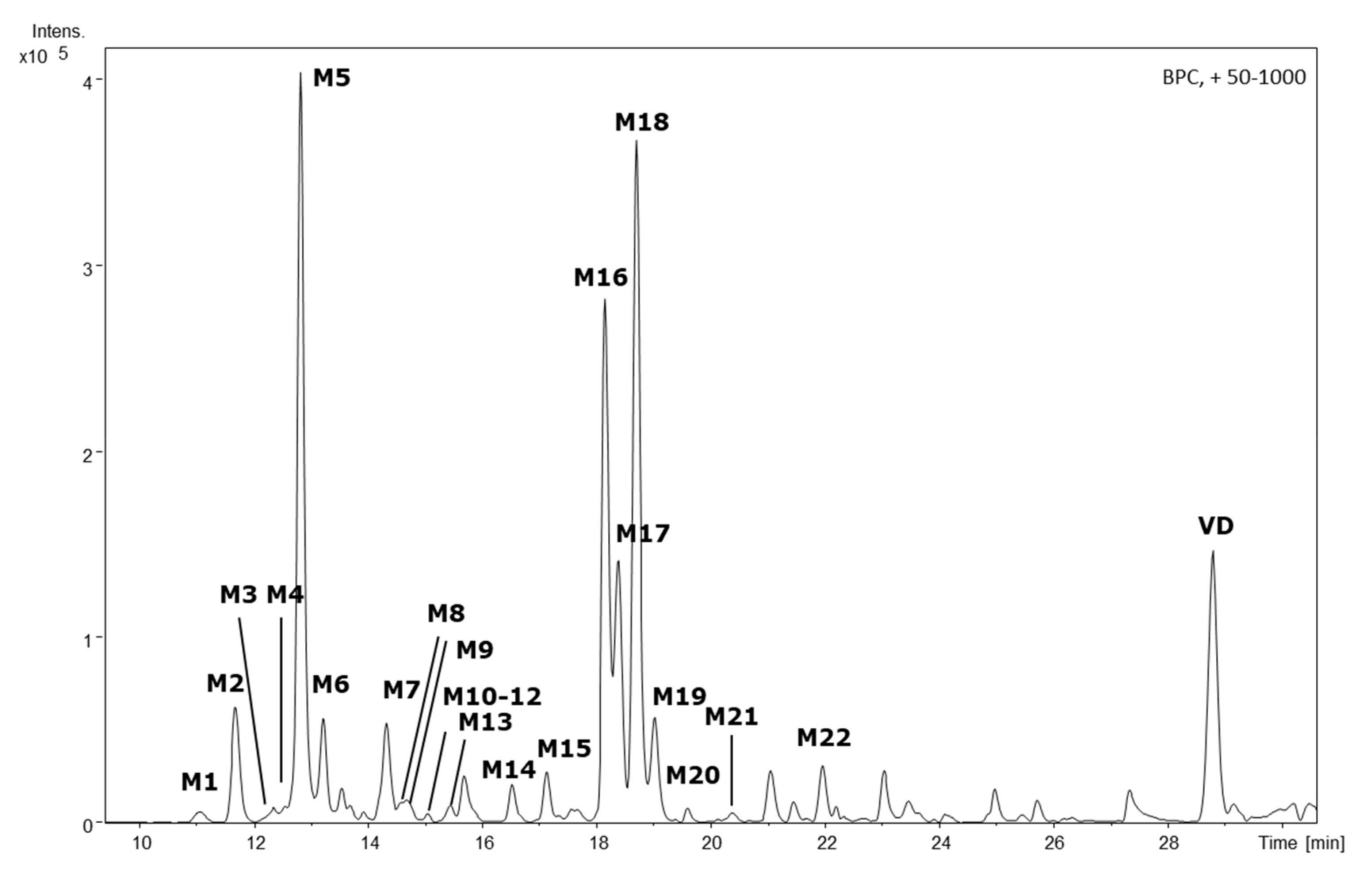
HPLC-QTOF-MS-derived base peak chromatogram of the HLM incubation supernatant featuring the parent compound vitetrifolin D (VD) and its phase I metabolites M1–M22. Experimental conditions HPLC: column: Zorbax Eclipse XDB-C18, 3 × 100 mm, 3.5 µm; mobile phase: A: H_2_O, B: acetonitrile, gradient: 0 min: 80% A, 5 min: 80% A, 8 min: 65% A, 35 min: 2% A; temp: 25 °C; flow rate 0.3 mL/min; injection volume:10 µL. HR-MS: ESI, positive mode; nebulizer gas (N_2_) 30.5 psi, dry gas (N_2_) 8.0 L/min at 220 °C, capillary voltage 4.5 kV; mass scan range: 50–1000 *m*/*z* at 1 Hz.

**Figure 2:**
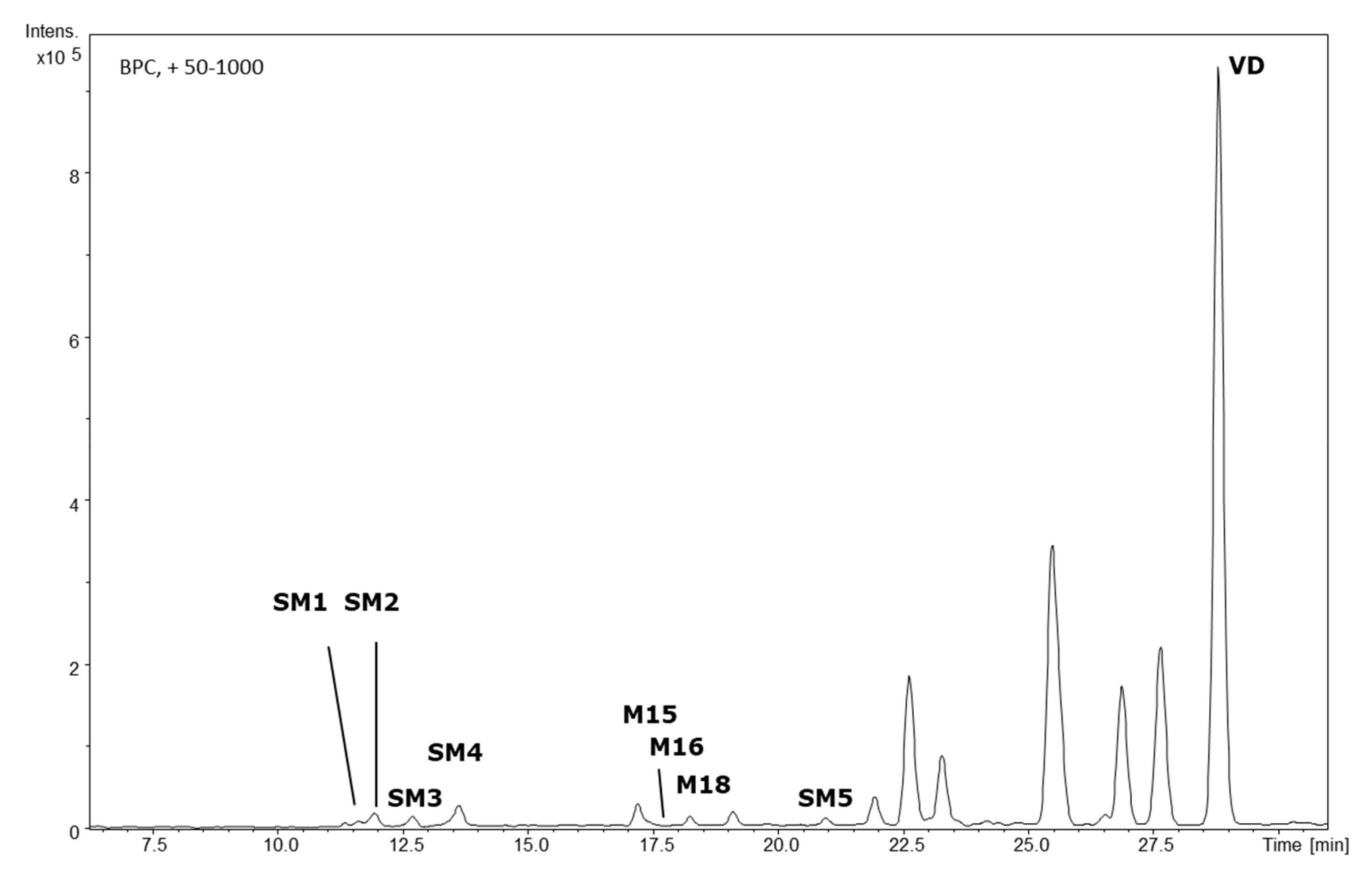
HPLC-QTOF-MS-derived base peak chromatogram of the S9 cell/UGT incubation supernatant featuring the parent compound, vitetrifolin D (VD), and its phase II metabolites SM1–SM5 and additional phase I metabolites. Experimental conditions as given in Figure 1.

With this approach, twenty-two of these features (M1–M22) were identified in the HLM cell culture supernatant and, therefore, tentatively assigned as phase I metabolites (Figure 1). In the S9/UGT cell culture supernatant, five additional metabolites were detected (SM1–SM5), in addition to three metabolites already found in HLM cell experiments (Figure 2). Tentatively, these analytes were assigned as phase II metabolites. In the S9/SULT cell culture, no additional metabolites, especially no sulfidations, were observable. For all twenty-seven identifiable metabolites, the accurate masses for the sodium and potassium adducts were obtained from the TOF-MS data. Elemental composition calculations for [M + Na]^+^ and [M + K]^+^ adducts, with a median mass accuracy of 1.7 ppm (5th percentile −4.1 ppm, 95th percentile 4.5 ppm), led to identical sum formulae for the respective [M]^+^ (Table 1).

### 2.3. Metabolite Identification

Analysis of the nominal mass shift between the metabolites and the parent compound, VD, allowed the analytes to be grouped depending on the metabolic reaction. In HLM incubations, oxidation of alkane or alkene carbons to alcohols or ketones predominated; in some cases, deacetylation reactions were observed. During incubation with S9 cells, glucuronidation reactions were additionally observable. Three of the twenty-two phase I metabolites found in the HLM cell experiments were also present in S9 cells, namely M15, M16, and M18.

#### 2.3.1. Single-Fold Oxidation 

The isobaric metabolites M16, M17, and M20, eluted in the retention time window from 18 min to 20 min in the extracted ion chromatogram (EIC), shared the molecular formula C_24_H_38_O_6_, which indicates a mass difference of +16 Da to VD (Figure S2). This indicates an oxidative metabolism event involving an alkane carbon center and leading to an additional hydroxy function in the VD scaffold. Alternatively, an epoxidation of a double bond would lead to an identical mass shift. Another metabolite (M22), eluted at 22 min, had a mass difference of only +14 Da to VD. The analysis of the sum formula C_24_H_36_O_6_ showed that, compared to VD, a double bond equivalent was gained. Hence, the assumed oxidation reaction either led to the formation of a carbonyl function (ketone) or was accompanied by a reduction step leading to an enol structure element.

#### 2.3.2. Twofold Oxidation

The isobaric metabolites M18 and M19 were eluted within 0.4 min at approximately 19 min retention time (Figure S3). Their molecular formula featured a mass difference of +34 Da to VD. This indicates that the twofold oxidative metabolic reaction (+32 Da) was accompanied by the reduction of an alkene moiety to an alkane (+2H). Hence, it can be assumed that M18/M19 are metabolites involving one of the two VD double bonds forming 1,2 diol moieties.

A mass shift of +32 Da to VD was associated with the isobaric metabolites M8, M12, M13, and M21. M8–M13 were eluted at around 15 min (time window of 1.2 min), significantly earlier than M18/M19. M21 was eluted shortly after this metabolite pair, at 20.3 min (Figure S3). The mass difference corresponds to the addition of two oxygen atoms, hence twofold oxidative metabolic transformation leads to these congeners. Either the two alkane carbons are hydroxylated or one of the oxygen substitutions involves a VD double bond, leading to an epoxide (which can subsequently be hydrolyzed to a vicinal diol).

Since epoxide formations do not lead to added hydrophilicity to the extent that is expected for a hydroxy function, it can be hypothesized that M21 with a chromatographic retention time similar to mono-hydroxylated metabolites features one hydroxy function and one epoxide function, whereas the other isobars (M8, M12, M13) are VD-diols. Metabolites with a mass shift of +30 Da corresponding to a twofold oxidation with the addition of a double bond equivalent e.g., a hydroxylation combined with a ketone formation, were not present in the reaction mixture.

#### 2.3.3. Threefold Oxidation

Almost all the early eluted metabolites (11 to 15 min) featured a positive mass shift corresponding to a threefold oxidation reaction (Figure S4). Five analytes (M1, M2 M3, M5, M6) showed a molecular weight gain of +50 Da compared to VD, which corresponded to the net loss of a double bond equivalent. This was in correspondence with threefold oxidation, in combination with reduction in one of the VD double bonds, resulting in a vicinal diol motif. Metabolites M4, M7, and M9 featured a mass shift of +48 Da compared to VD; hence, the number of double bond equivalents remained unchanged. If a double bond attack is part of the oxidative metabolism of these metabolites, an epoxide moiety and two hydroxy-functions are formed. Alternatively, threefold alkane oxidation takes place or the double bond is oxidized to a vicinal diol and, additionally, an alkane hydrocarbon atom is oxidized to a ketone.

#### 2.3.4. Hydrolysis

Only four of the twenty-two metabolites identified in the HLM experiments featured a molecular weight loss compared to VD. M15 (C_20_H_34_O_3_) showed the largest loss, with a reduction of −84 Da (C_4_H_4_O_2_), and the loss of two double bond equivalents. The chromatographic elution position of M15 (retention time 17.1 min) placed M15 between mono- and dehydroxylated VD metabolites (Figure S5). Hence, it can be hypothesized that M15 underwent di-deacetylation at positions 6 and 7, leading to a vicinal diol motif. Mono-deacetylation was not observed in the HLM incubation supernatants, but in the S9 cell incubation with metabolite SM5 (retention time 20.9 min), a mass difference of −42 Da to VD was shown, which corresponds to an acetate moiety loss.

Metabolites M10, M11, and M14 had a positive mass shift of 16 or 14 Da relative to M15, indicating that, in addition to the loss of two acetate moieties, an oxidative metabolic step took place. In the case of M11 and M14 (+16 Da), an aliphatic hydroxy function was introduced or a double bond oxidation took place, whereas M10 oxidation led to an additional ketone function in the scaffold.

#### 2.3.5. Glucuronidation

Analyte SM4, eluted at 13.6 min, showed +176 Da (C_6_H_8_O_6_) mass difference to VD (Figure S6). This molecular formula corresponds to a glucuronyl substituent. With only the C13 hydroxy function available as a substitution position, SM4 can be readily identified as vitetrifolin D 13-O-glucuronide. Analytes SM1, SM2, and SM3 were isobaric with a mass shift of +74 to VD and −100 to SM4. Since twofold deacetylation corresponds to a mass loss of −84 compared to VD (M15), it can be assumed that, in SM1–SM3, a di-deacetylation is followed by a glucuronidation step at one of the available hydroxy-functions (C6, C7, C13), accompanied by a dehydration reaction (−18 Da), transforming the vicinal 6,7-diol moiety in an enol structure element. Hence, two regio-isomers are possible, a 6-en-6,13-diol and a 6-en-7,13-diol. Consequently, mono-substitution with glucuronic acid (GlcA) led to the three isobaric glucuronide metabolites SM1–SM3. Di-glucuronide VD derivatives were not detectable in the S9 experiments. 

#### 2.3.6. Sulfation

The incubation of VD in the presence of PAPS did not yield any useful metabolic feature indicating the presence of VD-sulfates. Incubation times, VD concentration, and PAPS concentration were varied to confirm the negative finding.

### 2.4. HPLC-SPE-NMR Analysis

Since analysis by mass spectrometry allows for the structural characterization of metabolites beyond doubt only in selected cases, such as SM4, where the only available hydroxy function did undergo glucuronidation, and M15, where both acetyl-moieties were cleft off, utilization of NMR spectroscopy was inevitable. The complex mixture of isobaric metabolites made successful metabolite characterization via preparative analyte separation, followed by offline NMR, highly unlikely, as previous investigations proved [33,34,35]. Hence, the more sensitive and less cumbersome online approach via HPLC-SPE-NMR was pursued.

Due to the very limited amount of substance used in single HLM incubation experiments (100 nmol VD total, 80% transformed to >20 metabolites), incubations were repeated 17-fold to increase the absolute analyte amount in the HPLC-SPE-NMR samples. In addition to repeated metabolite generation, an offline SPE protocol was established to separate metabolite-containing fractions from the incubation matrix and to allow for further sample concentration prior to HPLC-SPE-NMR. While the analytical HPLC-DAD-QTOF-MS assay was optimized for monitoring the bioassay, the chromatographic separation in the HPLC-SPE-NMR setting needed optimization to provide optimal post LC metabolite peak trapping conditions onto SPE cartridges. To ensure the baseline separation of the metabolite peaks, a significantly longer and shallower gradient was designed and column overloading was strictly avoided. The trapping process of individual analytical peaks to individual SPE cartridges was repeated ten times to increase the analyte concentration in the subsequent NMR analysis. Analytes trapped on the SPE cartridges were dried with ambient temperature nitrogen and were stored in a nitrogen-flushed instrument cabinet until NMR analysis. All analytes were transferred to the spectrometer with deuterated acetonitrile (CD_3_CN), and a set of 1D and 2D NMR spectra was recorded. Signal and shift value assignment of proton and carbon atoms relied on these homonuclear and heteronuclear shift correlation experiments (DQF-COSY, NOESY; HSQC, HMBC) and on signal analysis of the ^1^H-NMR spectrum. High-resolution ^13^C NMR spectra were not recorded due to the limited sample amounts available.

**Scheme 2:**
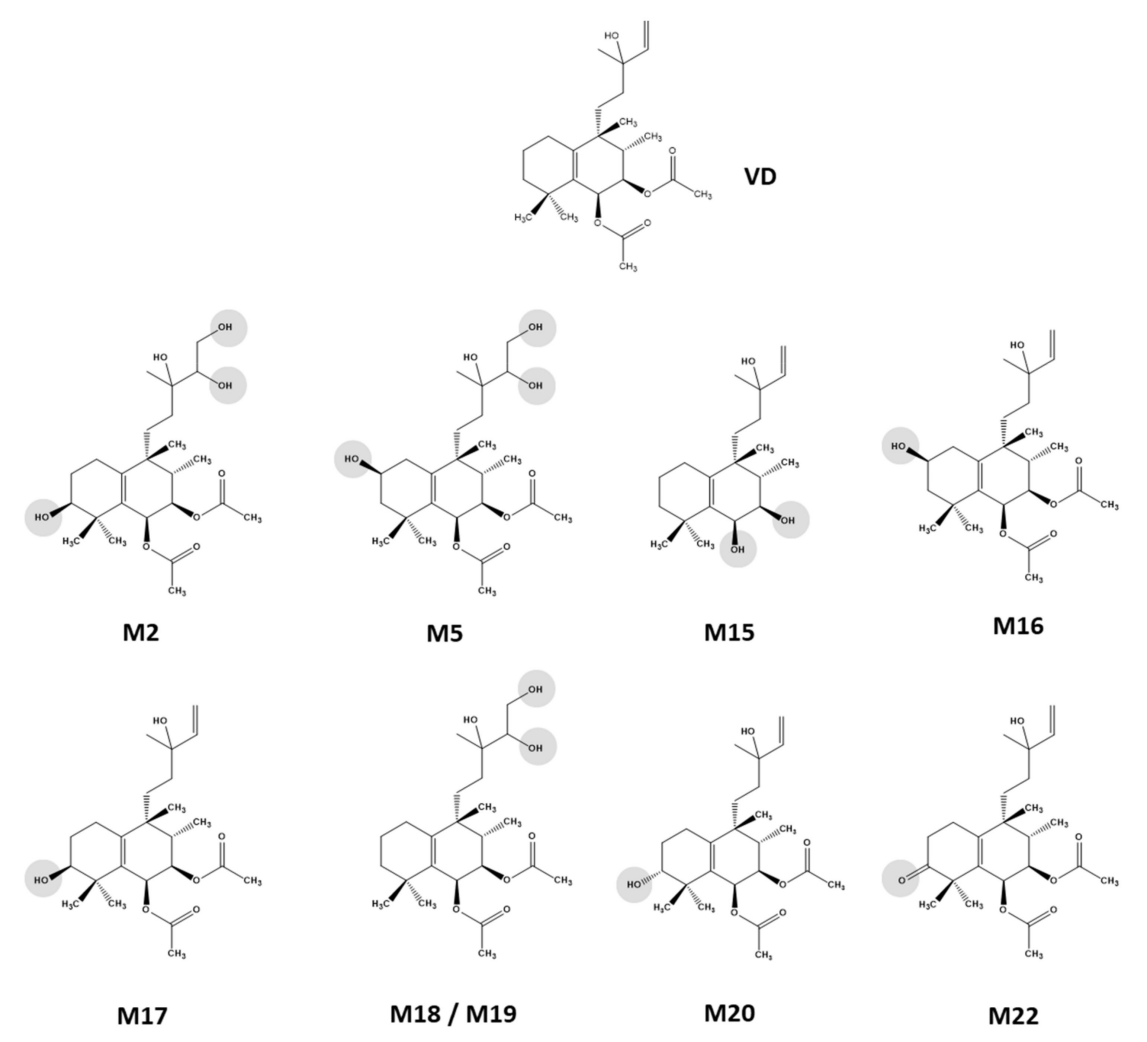
Vitetrifolin D (VD) phase I metabolites isolated from HLM incubations via HPLC-SPE-NMR and characterized by 1D and 2D NMR spectroscopy combined with sum formula generation by HR-mass spectrometry.

For nine VD metabolites, NMR-based structure elucidation was feasible from the recorded 1D and 2D NMR data (Scheme 2). The following paragraphs provide the details of this analysis block. Due to the small amount of substance available for the remaining thirteen metabolites, it was not possible to record a sufficient set of NMR spectra for structural characterization. Therefore, the substitution patterns could not be clearly determined for these analytes. For S9 cell incubations, no HPLC-SPE-NMR experiments were performed; hence, for SM1–SM5, structural information deduced from mass spectrometry data was not confirmed by NMR.

### 2.5. NMR-Based Structure Elucidation

#### 2.5.1. Vitetrifolin D (VD)

To aid in the structural elucidation of the VD-metabolites and to minimize the expectable (slight) shift value differences to spectra recorded in more conventional offline-NMR spectroscopy solvents such as CD_3_OD or CDCl_3_, a thorough analysis of VD (C_24_H_38_O_5_) NMR spectra in the HPLC-SPE-NMR solvent CD_3_CN was undertaken.

As expected, the ^1^H-NMR spectrum (Table S1) featured seven methyl group signals (six singlets and one doublet). Aided by HMBC correlation signals, two CH_3_ signals were assignable to acetate moieties and a C(CH_3_)_2_ group was readily identified. The ^1^H-NMR signal clusters of five aliphatic methylene groups featured very complex coupling patterns. HSQC-based shift correlation analysis unveiled that only one of these featured a significant proton anisotropy effect. One methylene group signal resonated significantly downfield; ^1^H/^13^C one- and multiple-bond shift correlation signals confirmed the presence of a vinyl group (C14, C15). Three aliphatic methine groups were identified from the HSQC spectrum, two with the characteristic downfield shift for oxygen substitution (C6, C7). The olefinic methine group was readily assignable to the vinyl function by DQF-COSY and HMBC correlation signals (Scheme S3). Only seventeen of the twenty-four carbon atoms could be characterized by signals in HSQC spectrum. The remaining seven signals were, therefore, assigned to quaternary atoms. Two of these signals were the carbonyl groups of the acetate functions; another atom was assigned to the C(CH_3_)_2_ group discussed above. All three were easily identified by HMBC contacts, starting from the methyl groups. By applying the same strategy, the quaternary sp^3^-hybridized centers C9 and C13 were also easily assigned. Signals for assignment of the sp^2^ hybridized bridgehead atoms linking halimane rings A and B were also found in the HMBC correlation signals of methyl groups C18, C19, and C20. Taken together, the NMR data confirmed the presence of a diterpenoid bicyclic structure with a side chain ending in a vinyl group. Three hydroxy functions were present—the two vicinal ones at the ring system were acetylated and the side chain moiety was unsubstituted. Combining the analysis of vicinal *^3^J_H,H_* coupling constants and homonuclear dipolar coupling NOESY cross peaks allowed for the assignment of the relative stereochemistry at the chiral carbon centers C6, C7, C8, and C9 as *rel*-6*S*,7*R*,8*S*,9*R* (Scheme S3). The configuration of the chirality center in the side chain (C13) remained unclear based on the gathered NMR data.

Since the absolute configurations were not available for VD or any other vitetrifolin type 8-*epi*-halim-5(10)-ene derivatives [26,36], the recently published absolute configuration of viterofolin H (= 1-hydroxy-vitetrifolin D; 1-OH-VD) must be interpreted with great caution [37]. The VD relative stereochemistry of C6–C9 based on NOE contacts and biosynthesis considerations is unquestioned, and hence can be confirmed from 1-OH-VD as 6S,7R,8S,9R. However, deriving the configuration of C13 from ^13^C-NMR shift value arguments seems questionable, as long as a putative C13 epimer is not compared under identical measurement conditions. Hence, we concluded that, with the current state of knowledge, the absolute configuration of the VD side chain could not be determined. Consequently, vitetrifolin D was elucidated as (6S,7R,8S,9R,13RS)-6,7-diacetoxy-5(10),14-halimadien-13-ol (Scheme 1).

#### 2.5.2. Metabolites with Onefold Oxidation (M16, M17, M20, M22)

**Figure 3:**
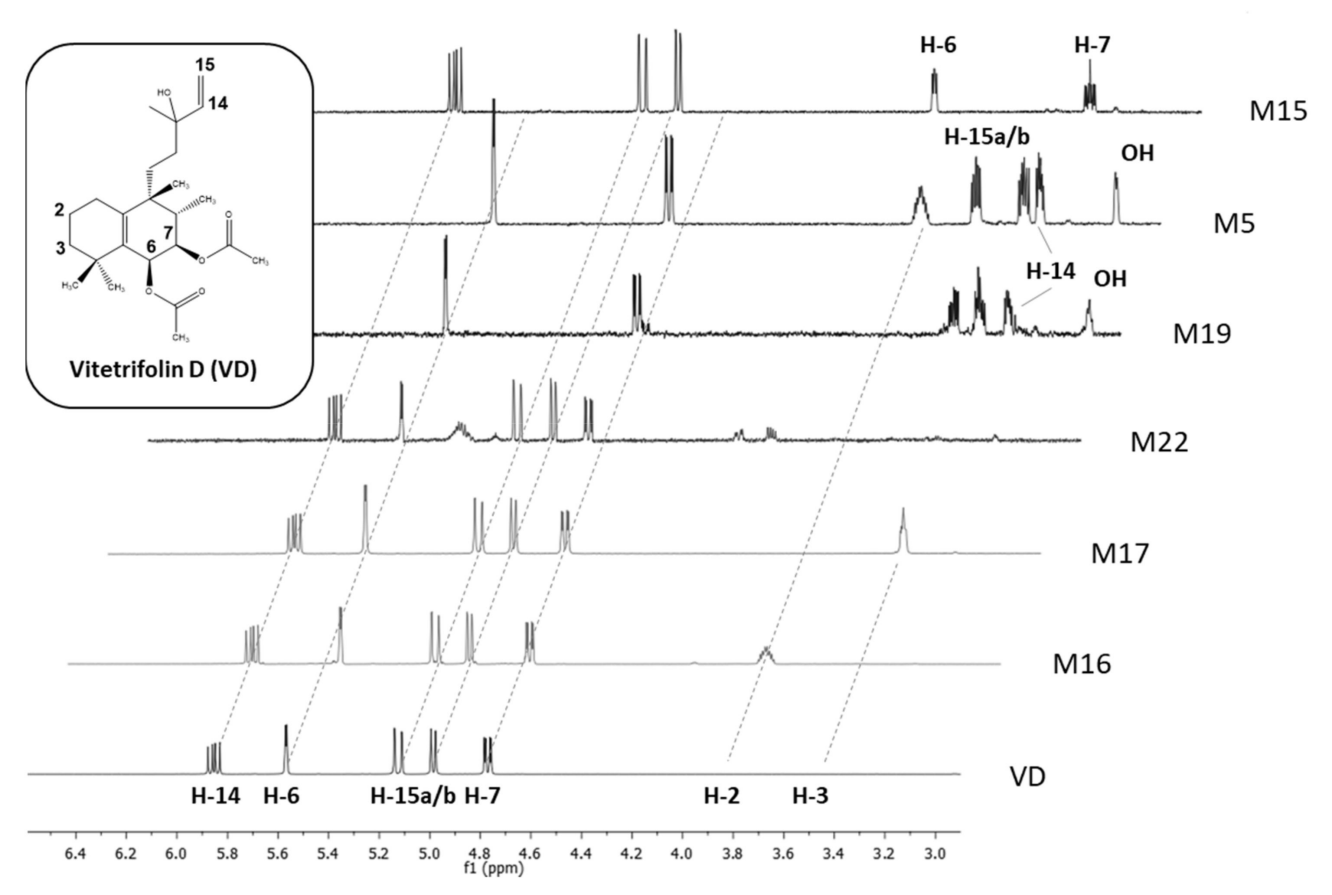
HPLC-SPE-NMR-recorded proton NMR spectra portion of vitetrifolin D (VD) and metabolites (600 MHz, solvent CD_3_CN), featuring signals of alkene protons and protons of hydroxylated alkane carbon centers. Inter-experimental shift value stability of structural features without changes in the chemical environment (e.g., vinyl group in VD, M15-M17, M22) allows for confirmation of its presence and understanding of its absence (M5, M19) as a structural change involving this structural motif.

**Figure 4:**
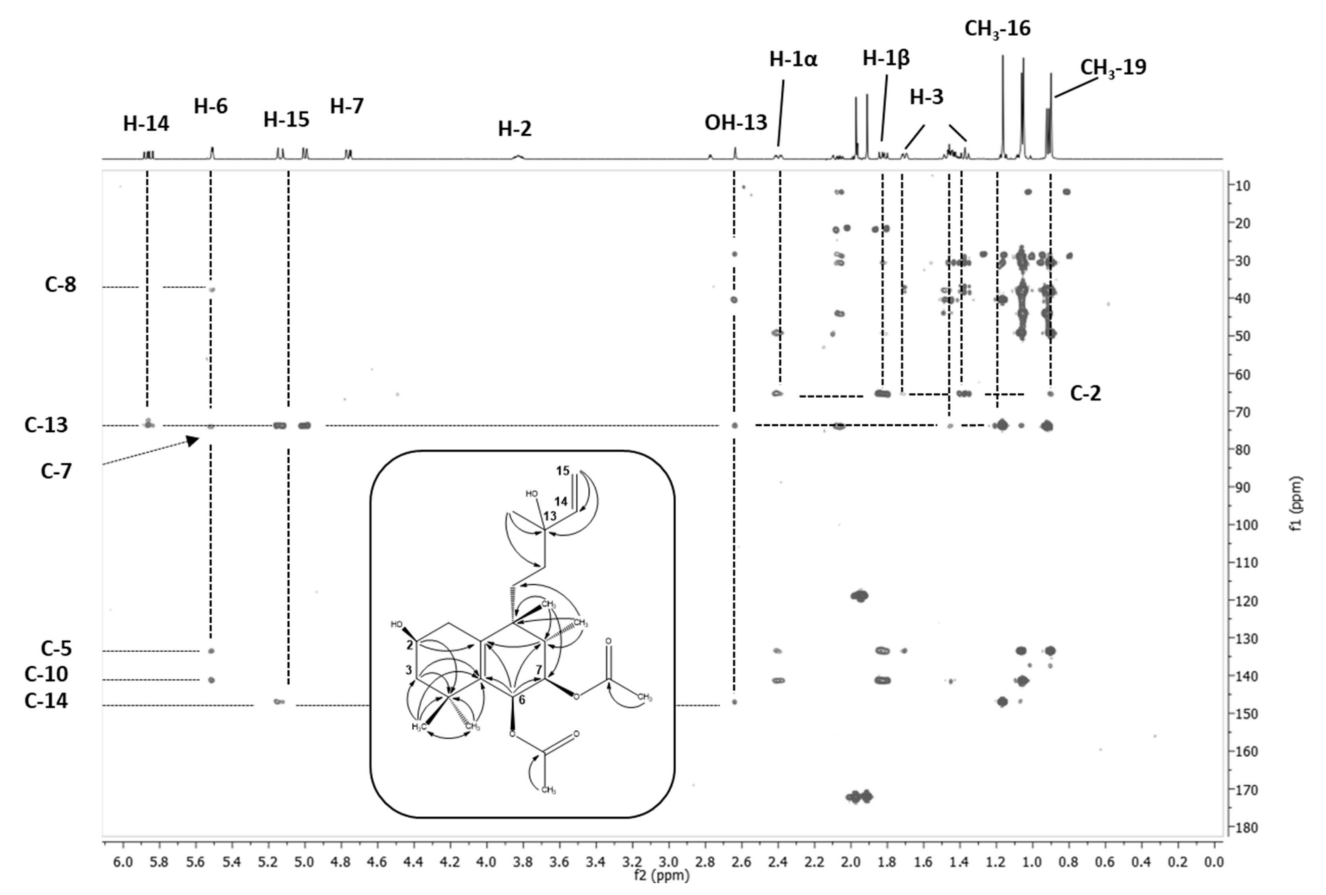
HPLC-SPE-NMR-based long-range shift correlation (HMBC) NMR spectrum of M16 (2β-OH VD) recorded in CD_3_CN at 300K. Experimental conditions: pulse program hmbcgplpndqf; 2K data points in the ^1^H domain, 256 increments in the ^13^C domain, zero filled to 1K. Recording of 96 transients per increment, acquisition time 11.5 h. The C-H correlation signals not only allowed for connection of the individual proton networks of the H-H correlation pattern derivable substructures, but also for unequivocal placement t of metabolic oxidation at the carbon position C2.

^1^H- and ^13^C-NMR spectra of all three metabolites with a mass difference of +16 Da to VD (M16, M17, M20) showed vinyl group signals (Figure 3). Hence, epoxidation of this double bond did not take place. HMBC correlation signals from the geminal methyl groups at C4 were in favor of a C5/C10 double bond. Consequently, no epoxidation took place and all congeners were hydroxy-VD derivatives. Analysis of the HMBC cross-peak pattern did unveil that M17 was hydroxylated at position C3, whereas M16 was hydroxylated at position C2 (Figure 4).

The hydroxylation position of M20, which was present in a much smaller amount in the metabolite mixture, was deduced by comparison with M16 and M17, as well as the 1-OH-VD known from the literature [37]. When VD was substituted at C1 or C2, the ^13^C shifts in methyl groups C18 and C19 were practically isobaric, while hydroxylation at position C3 triggered a clear shift anisotropy. M20 showed such a shift anisotropy, but not to the same extent as M17 (Tables S6 and S8). Since the coupling pattern of the CH(OH) proton was also clearly distinguishable from that of the a-terminal proton in M17 (two small coupling constants), M20 can be assumed to be the C3 epimer to M17. Molecular modeling of the two half-chair epimers did show that C3 was situated above the ring plane, bringing the 3β substituent (H or OH) into an axial position. The 3α substituent was in an equatorial position; both substituents at C2 showed an identical dihedral angle of approximate 30°, whereas the dihedral angles of the 3β substituent were notably different to the C2 substituents—one angle was approximately 30° and the other one approximately 180°. Hence, in M17, with H3 featuring two small and identical coupling constants, this proton was oriented equatorially and the OH function was in an axial position. M17 was, therefore, 3β-OH-VD, whereas M20 was 3α-OH-VD. The C2 configuration of M16 was confirmed as β-hydroxylated by the analysis of coupling constants of the C1, C2, and C3 protons and the presence of NOE contacts between the axially orientated methyl group C18 (substituent of C8), two axially orientated protons at C1 and C3, and one of the C4 methyl groups (Table S5). Consequently, M16 was identified as 2β-OH-VD. M22 showed a reduced mass difference of +14 Da compared to the hydroxylated metabolites, and an oxidation reaction with the formation of a double bond was assumed. The analysis of the NMR spectra allowed for the clear positioning of this functionality in order to position C3, since the HMBC contacts of both C4 methyl groups showed a strong correlation signal at δ_C_ = 215 ppm (Table S9). M22 was identified as 3-oxo-VD.

#### 2.5.3. Metabolites with Twofold Oxidation (M18, M19)

The NMR spectra of M19 lacked vinyl group signals, which were replaced by two signals (δ_H_ = 3.35 ppm and 3.56 ppm; δ_C_ = 76.8 ppm and 64.5 ppm), corresponding to hydroxylated sp^3^ hybridized carbon centers (Table S7). Since mass spectrometry of M19 featured a mass difference of +34 Da compared to VD, the replacement of the vinyl group at C14/C15 with a vicinal diol moiety was confirmed. The stereochemistry of the new chirality center at C14 was not determined. Hence, M19 was identified as 14,15-dihydro-14,15-dihydroxy-VD. Due to the lack of substance, the isobaric metabolite M18 could not be completely characterized by NMR. However, since the vinyl group was also replaced by a diol, it can be assumed that M18 was the C14 epimer to M19.

#### 2.5.4. Metabolites with Threefold Oxidation (M2, M5)

The NMR spectra of M2 and M5, both with a mass shift of +50 Da compared to VD, indicating that three oxygen functions were added and one double bond was lost in the course of the metabolic attack, featured vinyl group replacement by a vicinal diol. A thorough analysis of the shift correlation spectra placed the third oxygen at C2 (M2) and C3 (M5), respectively. Comparison with the NMR spectra of the singly hydroxylated derivatives M16 and M17 showed that identical stereochemical relationships were present for M2 and M5 (Tables S2 and S3). Hence M2 was 2β hydroxylated and M5 was 3α hydroxylated. Due to sample limitations, the structural characterization of the isobaric metabolites M1 and M3 was not possible, and it remains unclear if these substances were regio- or stereoisomers to M2 and M5.

#### 2.5.5. Metabolites with Hydrolysis of Acetate Groups (M15)

Compared to VD, the NMR spectra of M15 lacked acetate methyl group signals (Table S4). Furthermore, the protons at C6 and C7 significantly shifted to a higher field (approximately −2.5 ppm), indicating loss of the substituents (Figure 3). Since the mass spectra analysis showed a negative mass shift of −84 Da compared to VD, M15 was identified as 6,7-di-deacetyl-VD. The literature refers to this derivative as vitetrifolin I, isolated from *Vitex trifolia*. [36].

### 2.6. Analytes without NMR-Based Structural Characterization

Of the thirteen HLM-derived VD metabolites that lacked sufficient NMR data to allow for a tentative structural characterization, five were threefold oxidized (M1, M3, M4, M6, M7), five were twofold oxidized (M8, M9, M12, M13, M21), and three underwent di-deacetylation combined with onefold oxidation (M9, M10, M14). The data gathered on the eight characterized congeners suggest that hydroxylation/oxygenation occurred at C1-C3 or at the vinyl double bond. The latter one, leading to 1,2-diol moieties, was most likely a downstream event from an epoxidation reaction at a double bond. Whenever a mass difference of +16 was observed, either an alkene epoxidation or an alkane hydroxylation might have taken place. However, no epoxide was isolated by the online experiments, perhaps due to their instability in the reaction mixtures, since 1,2-diol derivatives were isolated and characterized (see above). Furthermore, no evidence of a C5/C10 double bond hydroxylation was detected. This might reflect that such backbone hydroxylation is accompanied by a loss of the structural integrity of the diterpene ring system. The five metabolites additionally found in S9 cells (SM1–SM5) were not further characterized by NMR spectroscopy, since their structure can be derived from the mass spectrometry data (see above).

## 3. Discussion

The present study was devoted to the question of whether it is possible to accompany metabolism studies for substances of limited availability by applying state-of-the-art separation technologies in such a way that metabolites can be characterized. A pharmacologically important lead substance of the well-characterized medicinal plant *Vitus agnus-castus* was selected for this purpose. It was shown that a large number of metabolites could be tentatively identified from the reaction broth of in vitro metabolism experiments of vitetrifolin D (VD), using HPLC-DAD-QTOF-MS and metabolite identification algorithms. For about half of these substances, the subsequent structural characterization by HPLC-SPE-NMR was successful; nine phase I VD metabolites were unequivocally characterized. Five phase II metabolites were characterized with HPLC-DAD-QTOF-MS down to the level of substitution isomers. Thus, it was shown that, by using substance-saving and highly sensitive analytical methods, the phase I and phase II metabolism of VD can be characterized in its basic features. Metabolic turnover proceeds through several oxidation steps and hydrolysis of the acetate residue is also used to hydrophilize the metabolites. Glucuronidation completes the metabolism. The presence of sulfation could not be observed in this study, possibly due to experimental limitations. The significance of this study lies in the fact that the amount of substance used (2 µmol) was so small that, with this approach, the human metabolism of pharmacologically active medicinal plant lead substances can be successfully studied in vitro. If this analytical approach is combined with experiments on the uptake of analytes in the organism, it is possible to test whether the secondary plant metabolites that are bioactive in in vitro experiments reach the target organism and survive metabolism by its detoxification machinery. Bioavailability assessment of VD is a substantive limitation of this study, as uptake experiments were not performed. The in vivo significance of in vitro metabolism experiments depends on whether VD can reach the target organism at all.

## 4. Materials and Methods

### 4.1. Chemicals and Reagents

Vitetrifolin D (VD) was isolated from a methanolic extract of *Vitex agnus-castus* fruits at the Department of Pharmacognosy (University of Innsbruck, Innsbruck, Austria) with a purity higher than 99.5%, determined by high-performance liquid chromatography with ultraviolet-diode array detection (UV-DAD) and NMR spectroscopy [38]. 

Pooled female human liver microsomes (HLMs) and S9 cells, NADPH regeneration system (NRS) cofactor solutions (solution A (NADP^+^ and G6P) and B (G6PDH), UGT reaction (UTGR) mix solution A (UDPGA) and B (Alamethicin), and adenosine 3′-phosphate 5′-phosphosulfate (PAPS) were purchased from Corning (Kaiserslautern, Germany). Analytical grade buffer reagents for potassium phosphate buffer were purchased from Merck (Darmstadt, Germany) and for Tris-buffer from Carl Roth GmbH Co. (Karlsruhe, Germany), respectively. To prepare VD stock solution, dimethyl sulfoxide (DMSO) (SeccoSolv^®^, Merck, Darmstadt, Germany) was used. HPLC-grade solvents acetonitrile and methanol were purchased from Merck (Darmstadt, Germany). Water for the HPLC mobile phase was purified onsite with a Sartorius Stedim, Arium 611UV (Sartorius, Vienna, Austria). Deuterated acetonitrile (CD_3_CN, 99.8%) for nuclear magnetic resonance (NMR) spectroscopy was purchased from Eurisotop (Gif-sur-Yvette, Cedex, France). All other chemicals were analytical grade and obtained from Merck (Darmstadt, Germany).

### 4.2. Preparation of the Vititrifolin D Stock Solution

To prepare the 2 mM VD stock solution, 17.4 mg of the analyte were dissolved in 1 mL dimethyl sulfoxide and stored in a cryovial at −80 °C until use.

### 4.3. Cellular Assay Incubation Conditions

To avoid multiple freeze–thaw cycles and maintain enzyme activity, HLM, S9 cells and all other required reagents were divided into single use fractions and stored at −80 °C until needed. HLM and S9 cell incubations were performed in 2 mL reaction vials (Eppendorf; Hamburg, Germany) on an Eppendorf ThermoMixer (Eppendorf, Germany) thermostatted to 37 °C. As controls, reagent blank incubations without cells were performed for each assay.

#### 4.3.1. HLM Phase I Metabolism Assay

The HLM incubation mixture was formed of 200 µL 500 mM potassium phosphate buffer (pH 7.4), 725 µL water, 50 µL NRS solution A, 10 µL NRS solution B, and 50 µL VD stock solution (100 µM final concentration). After 5 min pre-incubation of the reaction mixture at 37 °C, 25 µL (0.5 mg) of the HLM cells were added. For the assay blank the VD stock solution was replaced by DMSO. Incubations were stopped after 24 h by adding 200 µL of 5% acetic acid in acetonitrile. The stopped reaction mixture was shortly mixed by vortexing and kept on ice until centrifugation (10 min, 14,000× *g*) to separate the cell debris from the supernatant, which was transferred to HPLC vials and stored at −20 °C until analysis.

#### 4.3.2. S9 Fraction UDP-Glucuronosyltransferase (UGT) Assay

The S9-UGT incubation mixture was formed of 200 µL 500 mM potassium phosphate buffer (pH 7.4), 645 µL water, 80 µL UTGR solution A, 200 µL UTGR solution B, and 50 µL VD stock solution (100 µM final concentration). For the assay blank, the VD stock solution was replaced by DMSO. After 5 min pre-incubation of the reaction mixture at 37 °C, 25 µL (0.5 mg) of the S9 cells were added. Incubations were stopped after 24 h by adding 200 µL of 5% acetic acid in acetonitrile. The stopped reaction mixture was shortly mixed by vortexing and kept on ice until centrifugation (10 min, 14,000× *g*) to separate the cell debris from the supernatant, which was transferred to HPLC vials and stored at −20 °C until analysis.

#### 4.3.3. S9 Cell Fraction Sulfation Incubations

For sulfation, the S9 fraction (20 mg/mL) was incubated with 0.5 M Tris-buffer (pH 7.5), 1.01 mg/mL adenosine 3′-phosphate 5′-phosphosulfate (PAPS, 2 mM final concentration), and VD stock solution (100 µM final concentration). The assays were performed analogous to P450 and UGT incubations.

### 4.4. HPLC-DAD-QTOF-MS

HPLC-DAD-QTOF-MS analysis of samples from in vitro incubations were performed on an Agilent 1200 Rapid Resolution series HPLC instrument, equipped with a binary pump, column oven, 80 Hz photodiode array diode detector (monitoring wavelength for VD was 210 nm), and autosampler (Agilent, Waldbronn, Germany). For sample stability during measurements, the autosampler was set at 4 °C. Separations were performed on a Zorbax Eclipse XDB-C18 column (100 mm × 3.0 mm, 3.5 µm particle size; Agilent, Waldbronn, Germany), guarded with a security guard cartridge (4 mm × 2.0 mm, Phenomenex, Torrance, CA, USA). Gradient elution was performed using water (A) and acetonitrile (B) with the following gradients: 0 min: 80% A, 5 min: 80% A, 8 min: 65% A, 35 min: 2% A, 50 min: 2% A; post-time: 10 min: 80% A. The system was operated at a flow rate of 0.3 mL/min at 25 °C. Injection volume was 10 µL. 

A micrOTOF-Q II mass spectrometer equipped with an electrospray interface (ESI), operating in positive ionization mode (Bruker-Daltonics, Bremen, Germany) was coupled to the Agilent HPLC 1200 system, as described above. Source parameters (+ESI mode) were set as follows: capillary voltage 4.5 kV, dry gas 8.0 L/min at 220 °C, nebulizer gas 30.5 psi. The mass range was set to 50–1000, with a scan rate of 1 Hz. For the determination of exact masses, internal calibration at the beginning of each analysis (0.1 to 0.6 min) was performed using 10 mM sodium formate in isopropanol-water (1:1). The Metabolite Tools software package (Bruker Daltonics, Bremen, Germany) with MetabolitePredict Version 2.0 and MetaboliteDetect 2.0 was utilized to predict VD metabolites from HPLC-DAD-QTOF-MS-derived mass information.

### 4.5. HPLC-SPE-NMR

#### 4.5.1. Instrumentation

HPLC-SPE-NMR experiments were performed on a Bruker Avance II 600 MHz spectrometer (Bruker Biospin, Rheinstetten, Germany) equipped with a 30 µL flow probe head and hyphenated to an Agilent 1200 series instrument (Agilent, Waldbronn, Germany) equipped with a variable wavelength detector (VWD) and an autosampler via the Bruker/Spark Prospekt II Solid Phase Extraction Unit (Bruker Biospin, Rheinstetten, Germany), which was used to automatically trap eluting chromatographic peaks of interest on 10 mm × 2 mm Hysphere GP (General purpose) resin cartridges (Spark, Emmen, Netherlands) after a post-column addition of water using a Knauer K120 HPLC pump (Knauer, Berlin, Germany).

#### 4.5.2. Sample Preparation

To obtain sufficient sample material, the HLM incubation assay was repeated seventeen times. Individual supernatants were stored at −20 °C until further processing. To allow for metabolite enrichment, the supernatants were fractionated via offline solid phase extraction (SPE) (Strata^®^ C18-E, 55 µm, 500 mg/6 mL, Solid Phase Extraction, Phenomenex, Aschaffenburg, Germany). After loading individual samples on SPE-cartridges, a gradient step elution (6 mL/step) was performed using water (A) and acetonitrile (B) mixtures with 10% step height. The collected fractions were analyzed with the aforementioned HPLC-DAD-QTOF-MS assay. Prior to HPLC-SPE-NMR analysis, fractions containing VD or putative VD metabolites were pooled, dried down under reduced pressure, and reconstituted in 1 mL methanol.

#### 4.5.3. HPLC-SPE-NMR Analysis

Chromatographic separations in the HPLC-SPE-NMR setup were performed on a Phenomenex Hydro RP-18 column (150 mm × 4.6 mm, 4 µm particle size, Phenomenex, Aschaffenburg, Germany) guarded with a security guard cartridge (4 mm × 2.0 mm, Phenomenex, Aschaffenburg, Germany) with a solvent gradient of H_2_O (A) and acetonitrile (B) at a flow rate of 0.8 mL/min, injection volume of 10 µL, and a recorded wavelength at 210 nm. Solvent gradients were optimized to allow for baseline separation for the analytes of interest. For SPE fractions exceeding 30% ACN: 0 min: 99% A, 11 min: 99% A, 11.5 min: 70% A, 60 min: 50% A, 100 min: 2% A for 20 min, post-time: 20 min: 99% A, the oven temperature was set to 50 °C. For SPE fractions less than or equal to 30% ACN: 0 min: 99% A, 11 min: 99% A, 11.5 min: 80% A, 60 min: 50% A, 80 min: 2% A for 20 min, post time: 20 min: 99% A, the oven temperature was 60 °C.

Peak trapping on the SPE device was triggered by the UV signal in the LC domain, and repeated tenfold for each HPLC peak of interest. A fivefold volume (4 min/min) of water was added to the LC effluent to allow for trapping of the peaks onto the SPE cartridges. Trapped LC peaks were dried for 35 min in a stream of nitrogen and eluted with 245 µL deuterated acetonitrile (CD_3_CN) in the probe of the NMR spectrometer. For 1D and 2D NMR experiments, standard pulse sequences provided by the spectrometer manufacturer were used. All spectra were recorded at 300 K and referenced to residual solvent peaks (δ_H_ 1.94 ppm and δ_C_ 1.24 ppm for CD_3_CN). Typical experimental conditions were: 1D ^1^H-NMR: pulse program lc1pnf2 ns = 512, 32 K data points, recording time 30 min. The 2D homonuclear shift correlations were as follows: COSY: pulse program cosygpmfqf, 4 K data points in the ^1^H domain, 400 increments, zero filled to 2 K, 16 transients per increment, recording time 4.5 h; TOCSY: pulse program mlevph, 2 K data points in the ^1^H domain, 256 increments, zero filled to 2 K, 32 transients per increment, recording time 5 h; ROESY: pulse program: roesyph, 2 K data points in the ^1^H domain, 128 increments, zero filled to 1 K, 128 transients per increment, recording time 11 h. The 2D heteronuclear shift correlations were as follows: HSQC: pulse program hsqcedetgpsisp2.2, 2 K data points in the ^1^H domain, 256 increments, zero filled to 1 K, 64 transients per increment, recording time 7.5 h; HMBC: pulse program hmbcgplpndqf, 2 K data points in the ^1^H domain, 128 increments, zero filled to 1 K, 192 transients per increment, recording time 11.5 h.

## 5. Conclusions

In the present study, in vitro biotransformation of VD (Scheme 1) in HLMs and the S9 cell fraction from liver tissue was performed. A total of twenty-seven phase I and phase II metabolites, mostly from oxidation reactions but also from acetate hydrolysis or glucuronidation reaction, were tentatively identified by chemometric means via their sum formulae, derived from HPLC-DAD-QTOF-based mass spectrometry data. Isolation from the HLM incubation broth by offline SPE enrichment followed by online HPLC-SPE-NMR was pursued for twenty-two of these congeners. The peaks of interest in the separation domain of analytical chromatography were isolated online after HPLC separation on SPE material, and further transferred in NMR solvent to the online probe head of the spectrometer. For nine of these metabolites the subsequent recording of the NMR spectra allowed for structural information to be obtained that was sufficient for structural characterization (Scheme 2). Since the preparative isolation of the metabolites was avoided, the substance input was much lower than in conventional strategies. The study did prove that the use of hyphenated instrumental analysis methodologies allows for the successful performance of in vitro metabolism studies, even if the availability of substances is very limited.

## Figures and Tables

**Table 1:** Tentative or HPLC-SPE-NMR-confirmed metabolites of vitetrifolin D (VD) formed in HLM (M1–M22), and additionally in S9 (SM1–SM5), cells. Analytes are sorted according to their retention times in the HPLC-DAD-QTOF-MS assay.

Compound	Retention Time [min]	[M + Na]^+^ Experimental [*m/z*]	[M + Na]^+^ Accuracy [ppm]	[M + K]^+^ Experimental [*m/z*]	[M + K]^+^ Accuracy [ppm]	Elemental Composition	Mass Shift to VD [u]	Tentative Type of Metabolism ^a^	Substituents by NMR ^b^
M1	11.1	479.2574	+2.4	495.2319	−1.7	C_24_H_40_O_8_	+50	1 * OH, 2 * OH + H	n.i.
M2	11.6	479.2581	−2.0	495.2336	+3.8	C_24_H_40_O_8_	+50	1 * OH, 2 * OH + H	OH @ 3β,14,15
M3	12.2	479.2575	−0.9	495.2337	+3.6	C_24_H_40_O_8_	+50	1 * OH, 2 * OH + H	n.i.
M4	12.4	477.2446	+2.7	493.2189	+1.9	C_24_H_38_O_8_	+48	3 * OH or 2 * OH and 1 * Ep or 1 * = O and 2 * OH + H	n.i.
M5	12.9	479.2572	−0.1	495.2316	−1.0	C_24_H_40_O_8_	+50	1 * OH, 2 * OH + H	OH @ 2β,14,15
M6	13.2	479.2575	−0.7	495.2321	−2.0	C_24_H_40_O_8_	+50	1 * OH, 2 * OH + H	n.i.
M7	14.3	477.2465	−1.3	493.2161	−1.4	C_24_H_38_O_8_	+48	3 * OH or 2 * OH and 1* Ep or 1 * = O and 2* OH + H	n.i.
M8	14.5	461.2456	+2.1	477.2246	−8.5	C_24_H_38_O_7_	+32	2 * OH or 1 * OH and 1 * Ep	n.i.
M9	14.7	477.2360	−6.2	493.2156	−0.3	C_24_H_38_O_8_	+48	3 * OH or 2 * OH and 1 * Ep or 1 * = O and 2 * OH + H	n.i.
M10	15.0	359.2121	+7.4	375.2006	−0.3	C_20_H_32_O_4_	−70	2 * deAc and 1 * = O,	n.i.
M11	15.1	361.2280	−6.2	377.2057	−3.4	C_20_H_34_O_4_	−68	2 * deAc and 1 * OH or 1 * Ep	n.i.
M12	15.5	461.2453	+2.8	477.2204	+0.2	C_24_H_38_O_7_	+32	2 * OH or 1 * OH and 1 * Ep	n.i.
M13	15.7	461.2455	+2.2	477.2214	−1.8	C_24_H_38_O_7_	+32	2 * OH or 1 * OH and 1 * Ep	n.i.
M14	16.5	361.2345	+1.2	377.2075	+3.6	C_20_H_34_O_4_	−68	2 * deAc and 1 * OH or 1 * Ep	n.i.
M15	17.1	345.2359	+3.7	361.2129	+2.9	C_20_H_34_O_3_	−84	2 * deAc	deAc @ 6,7
M16	18.1	445.2522	−1.2	461.2263	−1.5	C_24_H_38_O_6_	+16	1 * OH or 1 * Ep	OH @ 2β
M17	18.4	445.2543	+4.0	461.2285	+3.2	C_24_H_38_O_6_	+16	1 * OH or 1 * Ep	OH @ 3β
M18	18.7	463.2652	+3.1	479.2388	+3.7	C_24_H_40_O_7_	+34	2 * OH + H	n.i.
M19	19.0	463.2648	+3.9	479.2391	+3.1	C_24_H_40_O_7_	+34	2 * OH + H	OH @ 14,15
M20	19.6	445.2573	−2.8	461.2306	−1.3	C_24_H_38_O_6_	+16	1 * OH or 1 * Ep	OH @ 3α
M21	20.3	461.2454	+2.6	477.2211	−1.3	C_24_H_38_O_7_	+32	2 * OH or 1 * OH and 1 * Ep	n.i.
M22	22.0	443.2394	+2.3	459.2132	+2.5	C_24_H_36_O_6_	+14	1 * = O	= O @ 3
SM1	11.6	503.2591	+4.8	519.2348	+1.4	C_26_H_40_O_8_	+74	2 * deAc, 1 * deHy 1 * GlcA	n.i.
SM2	11.9	503.2609	+0.6	519.2356	+4.2	C_26_H_40_O_8_	+74	2 * deAc, 1 * deHy 1 * GlcA	n.i.
SM3	12.7	503.2608	+0.8	519.2346	+0.8	C_26_H_40_O_8_	+74	2 * deAc, 1 * deHy 1 * GlcA	n.i.
SM4	13.6	605.2946	+2.2	621.2672	+0.1	C_30_H_46_O_11_	+176	1 * GlcA	n.i.
SM5	20.9	387.2489	+4.4	403.2225	+4.9	C_22_H_36_O_4_	−42	1 * deAc	n.i.
VD	28.8	429.2597	+3.4	445.2339	+2.7	C_24_H_38_O_5_	0	parent compound	-

^a^ OH, hydroxylation of alkane; OH + H, hydroxylation of alkene; = O, oxidation of alkane; Ep, oxidation of alkene (epoxide formation); deAc, deacetylation; deHy, dehydration; GlcA, glucuronidation; 1 *, onefold; 2 *, twofold; 3 *, threefold. ^b^ Position numbering according to Scheme 1; n.i., not identified

## Data Availability

Raw data to the data presented in this study are available on request from the corresponding author. The data are not publicly available due to logistic reasons.

## Supplementary Materials

The following are available online at https://www.mdpi.com/article/10.3390/metabo11080529/s1, Scheme S1: Workflow sketch for the hyphenate instrument setups HPLC-DAD-QTOF-MS and HPLC-SPE-NNR utilized in this study, Scheme S2: HPLC-DAD-QTOF-MS-derived mass spectra of 2β-OH-VD (M16) and VD, Scheme S3: HPLC-SPE-NMR (1H (600 MHz)/13C (150 MHz), CD3CN)-derived correlations of VD: (a) multi-bond HMBC correlations (arrows) and COSY-correlations (red bonds); (b) NOESY correlation contacts, Figure S1: HLM incubation experiment with 100 µM VD. Base peak chromatograms (BPCs) of different sampling timepoints showing a stable metabolite profile after 24 h incubation time, Figure S2: HPLC-DAD-QTOF-MS-derived extracted ion chromatograms (EICs) for onefold oxidized metabolites generated in the HLM incubation experiment with 100 µM VD, Figure S3: HPLC-DAD-QTOF-MS-derived extracted ion chromatograms (EICs) for twofold oxidized metabolites generated in the HLM incubation experiment with 100 µM VD, Figure S4: HPLC-DAD-QTOF-MS-derived extracted ion chromatograms (EICs) for threefold oxidized metabolites generated in the HLM incubation experiment with 100 µM VD, Figure S5: HPLC-DAD-QTOF-MS-derived extracted ion chromatograms (EICs) for hydrolyzed metabolites generated in the HLM incubation experiment with 100 µM VD, Figure S6: HPLC-DAD-QTOF-MS-derived extracted ion chromatograms (EICs) for glucuroniated metabolites generated in the S9-UGT incubation experiment with 100 µM VD, Table S1: 1H (600 MHz)- and 13C (150 MHz) NMR data of VD (CD3CN), Table S2: 1H (600 MHz)- and 13C (150 MHz) NMR data of M2 (CD3CN), Table S3: 1H (600 MHz)- and 13C (150 MHz) NMR data of M5 (CD3CN), Table S4: 1H (600 MHz)- and 13C (150 MHz) NMR data of M15 (CD3CN), Table S5: 1H (600 MHz)- and 13C (150 MHz) NMR data of M16 (CD3CN), Table S6: 1H (600 MHz)- and 13C (150 MHz) NMR data of M17 (CD3CN), Table S7: 1H (600 MHz)- and 13C (150 MHz) NMR data of M19 (CD3CN), Table S8: 1H (600 MHz)- and 13C (150 MHz) NMR data of M20 (CD3CN), Table S9: 1H (600 MHz)- and 13C (150 MHz) NMR data of M22 (CD3CN).
