# Supplementary material for: Combining HPLC-DAD-QTOF-MS and HPLC-SPE-NMR to Monitor In Vitro Vitetrifolin D Phase I and II Metabolism
Source: Metabolites. 2021 Aug 9;11(8):529. doi: 10.3390/metabo11080529 (PMC8400717; doi:10.3390/metabo11080529)
Supplement: Supplementary file 1 [file metabolites-11-00529-s001.zip › metabolites-1281048-supplementary.pdf]

Supplement

Combining HPLC-DAD-QTOF-MS and LC-SPE-NMR to Monitor In Vitro Vitetrifolin D Phase I and II Metabolism

Sonja Sturm *, †, Christina Högner †, Christoph Seger and Hermann Stuppner

Institute of Pharmacy/Pharmacognosy, CCB - Centrum of Chemistry and Biomedicine, University of Innsbruck, Innrain 80-82, A-6020 Innsbruck, Austria
* Correspondence: sonja.sturm@uibk.ac.at; Tel.: +43 512 507 58401
† These authors contributed equally to this work.

Description of supporting material:

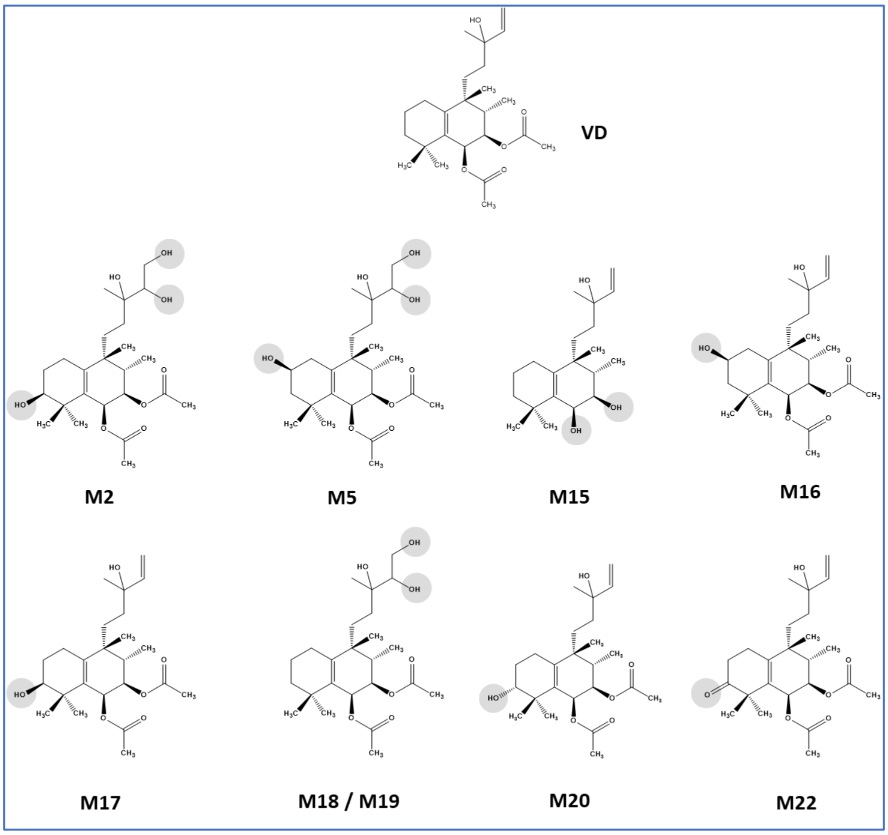

Scheme S1: Workflow sketch for the hyphenate instrument setups HPLC-DAD-QTOF-MS and HPLC-SPE-NNR utilized in this study.
Scheme S2: HPLC-DAD-QTOF-MS derived mass spectra of 2β-OH-VD (M16) and VD.
Scheme S3: LC-SPE-NMR (^{1}H (600 MHz) / ^{13}C (150 MHz), CD_3CN) derived correlations of VD: (**a**) multi-bond HMBC correlations (arrows) and COSY-correlations (red bonds); (**b**) NOESY correlation contacts.
Figure S1: HLM incubation experiment with 100 µM VD. Base peak chromatograms (BPCs) of different sampling timepoints showing a stable metabolite profile after 24 h incubation time.
Figure S2: HPLC-DAD-QTOF-MS derived extracted ion chromatograms (EICs) for single fold

oxidized metabolites generated in the HLM incubation experiment with 100 μM VD.
Figure S3: HPLC-DAD-QTOF-MS derived extracted ion chromatograms (EICs) for twofold oxidized metabolites generated in the HLM incubation experiment with 100 μM VD.
Figure S4: HPLC-DAD-QTOF-MS derived extracted ion chromatograms (EICs) for threefold oxidized metabolites generated in the HLM incubation experiment with 100 μM VD.
Figure S5: HPLC-DAD-QTOF-MS derived extracted ion chromatograms (EICs) for hydrolyzed metabolites generated in the HLM incubation experiment with 100 μM VD.
Figure S6: HPLC-DAD-QTOF-MS derived extracted ion chromatograms (EICs) for glucuroniated metabolites generated in the S9-UGT incubation experiment with 100 μM VD.
Table S1: ^{1}H (600 MHz)- and ^{13}C (150 MHz) NMR-Data of VD (CD_3CN).
Table S2: ^{1}H (600 MHz)- and ^{13}C (150 MHz) NMR-data of M2 (CD_3CN).
Table S3: ^{1}H (600 MHz)- and ^{13}C (150 MHz) NMR-data of M5 (CD_3CN).
Table S4: ^{1}H (600 MHz)- and ^{13}C (150 MHz) NMR-data of M15 (CD_3CN).
Table S5: ^{1}H (600 MHz)- and ^{13}C (150 MHz) NMR-data of M16 (CD_3CN).
Table S6: ^{1}H (600 MHz)- and ^{13}C (150 MHz) NMR-data of M17 (CD_3CN).
Table S7: ^{1}H (600 MHz)- and ^{13}C (150 MHz) NMR-data of M19 (CD_3CN).
Table S8: ^{1}H (600 MHz)- and ^{13}C (150 MHz) NMR-data of M20 (CD_3CN).
Table S9: ^{1}H (600 MHz)- and ^{13}C (150 MHz) NMR-data of M22 (CD_3CN).

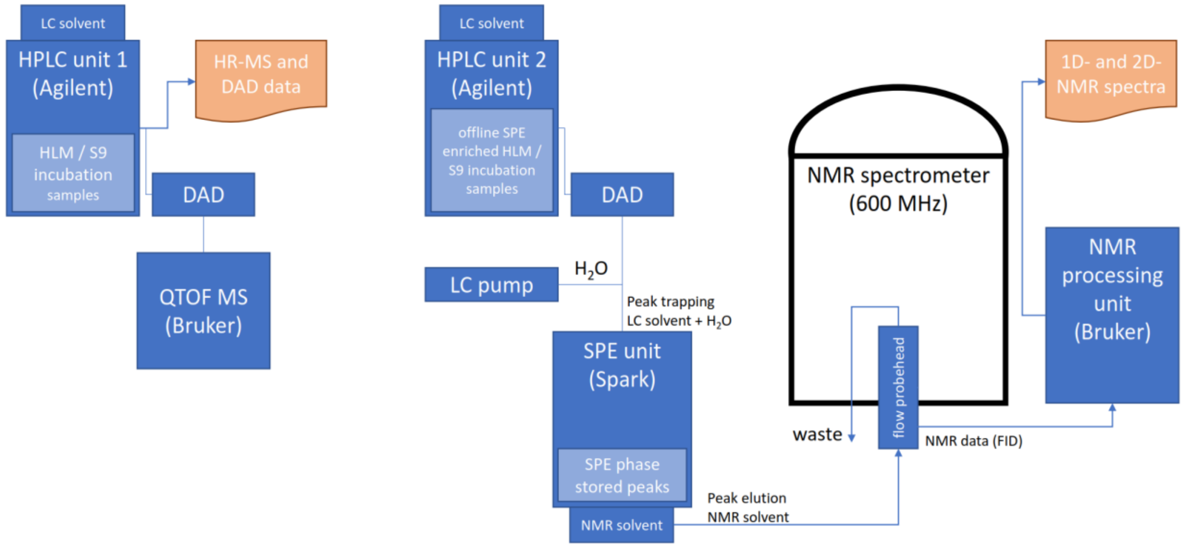

Scheme S1: Workflow sketch for the hyphenate instrument setups HPLC-DAD-QTOF-MS and HPLC-SPE-NNR utilized in this study.

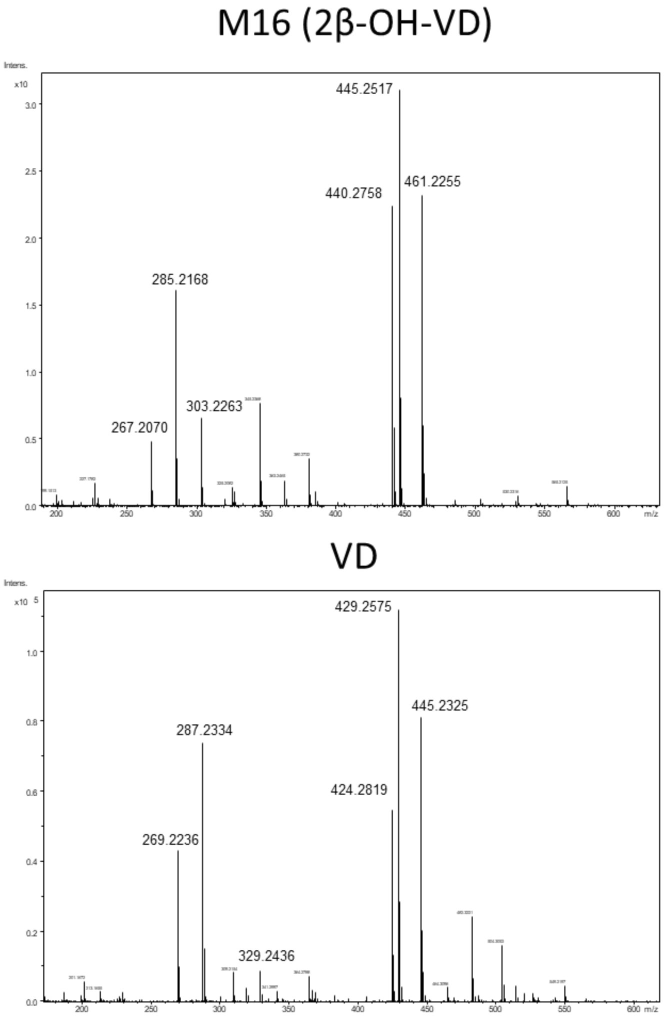

Scheme S2: HPLC-DAD-QTOF-MS derived mass spectra of 2β-OH-VD (M16) and VD.

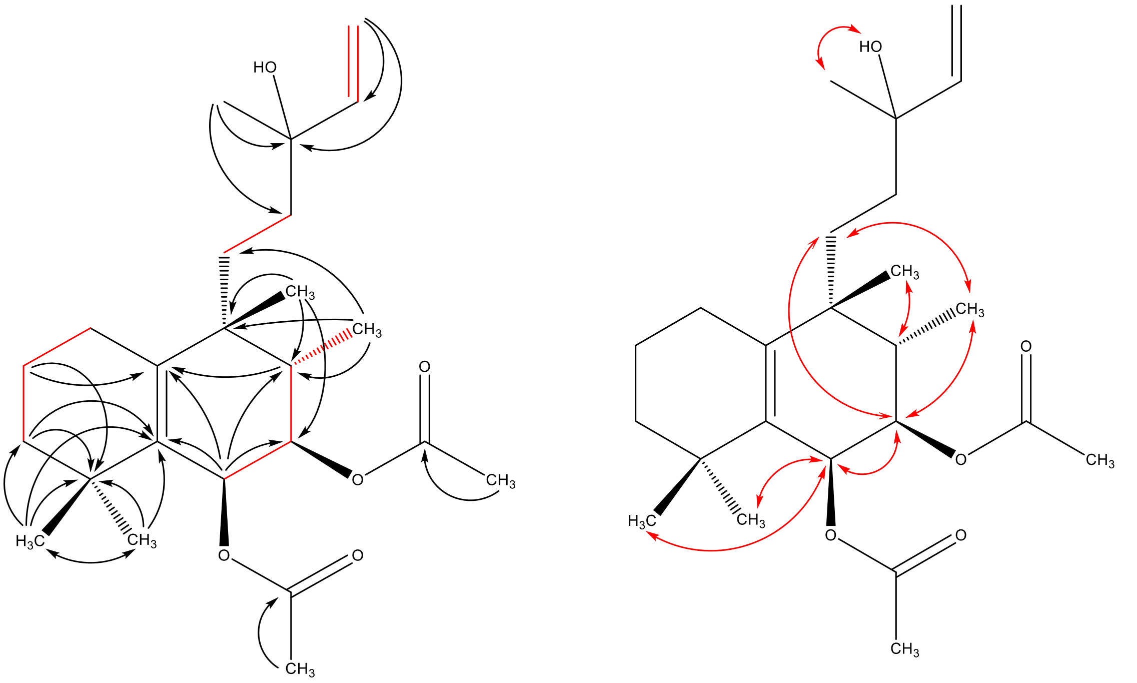

(**a**) (**b**)

Scheme S3: LC-SPE-NMR (^{1}H (600 MHz) / ^{13}C (150 MHz), CD_3CN) derived correlations of VD: (**a**) multi-bond HMBC correlations (arrows) and COSY-correlations (red bonds); (**b**) NOESY correlation contacts.

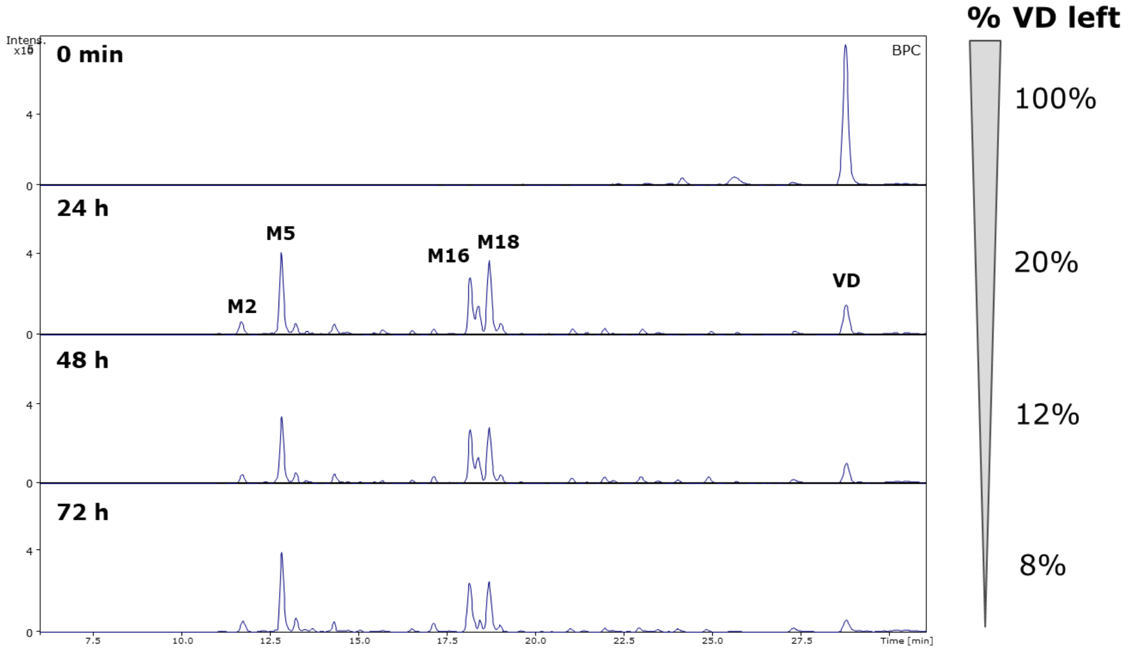

Figure S1: HLM incubation experiment with 100 µM VD. Base peak chromatograms (BPCs) of different sampling timepoints showing a stable metabolite profile after 24 h incubation time.

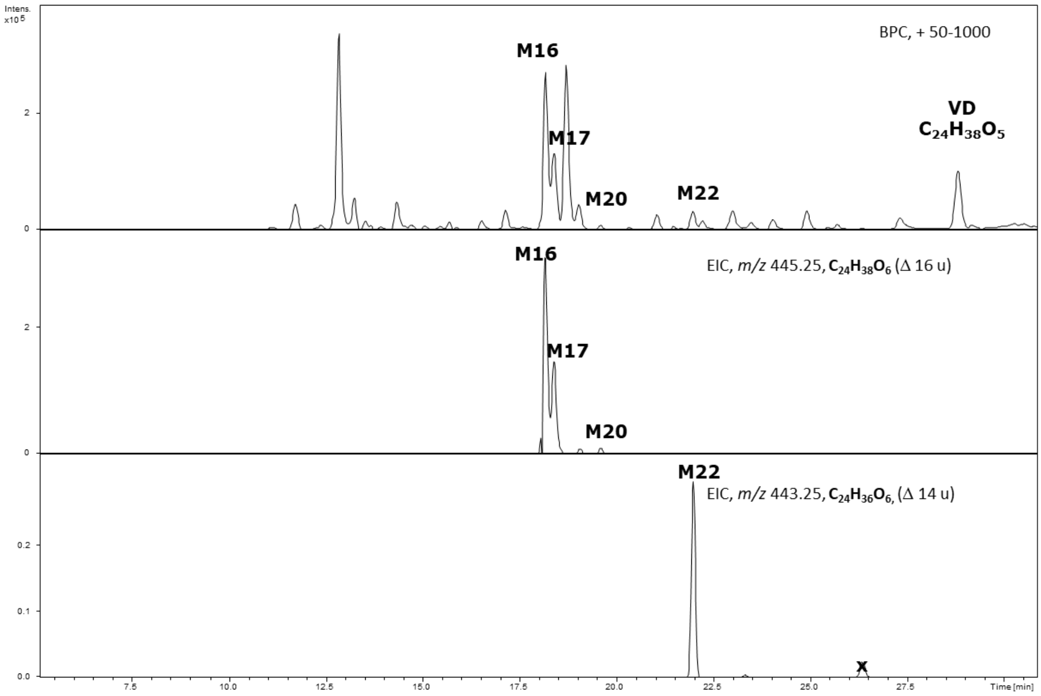

Figure S2: HPLC-DAD-QTOF-MS derived extracted ion chromatograms (EICs) for single fold oxidized metabolites generated in the HLM incubation experiment with 100 μM VD.

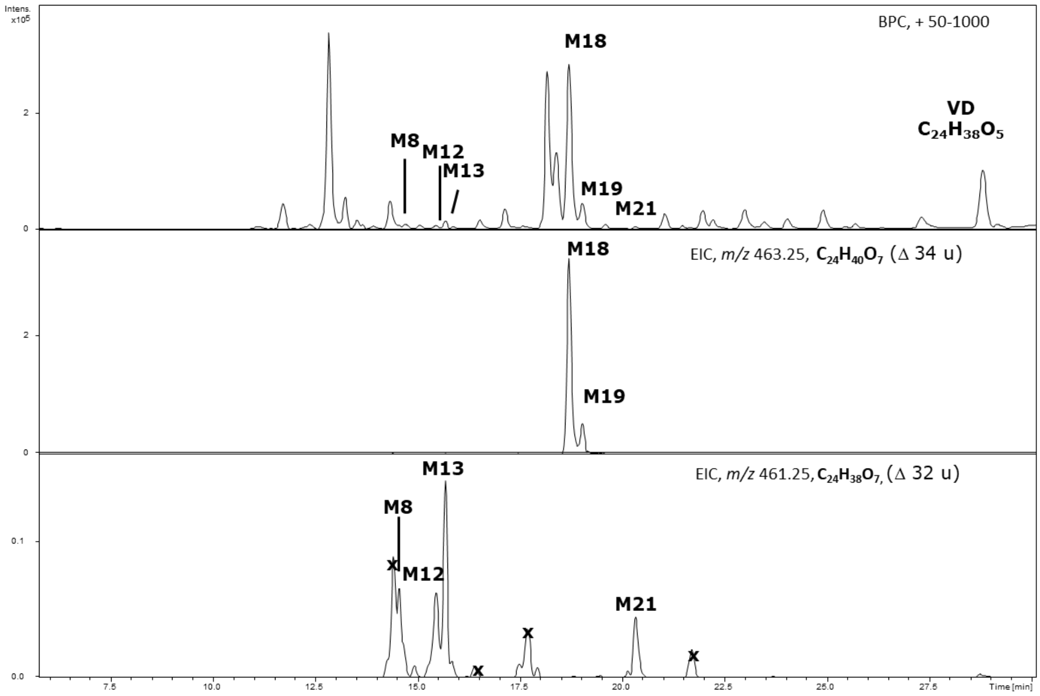

Figure S3: HPLC-DAD-QTOF-MS derived extracted ion chromatograms (EICs) for twofold oxidized metabolites generated in the HLM incubation experiment with 100 μM VD.

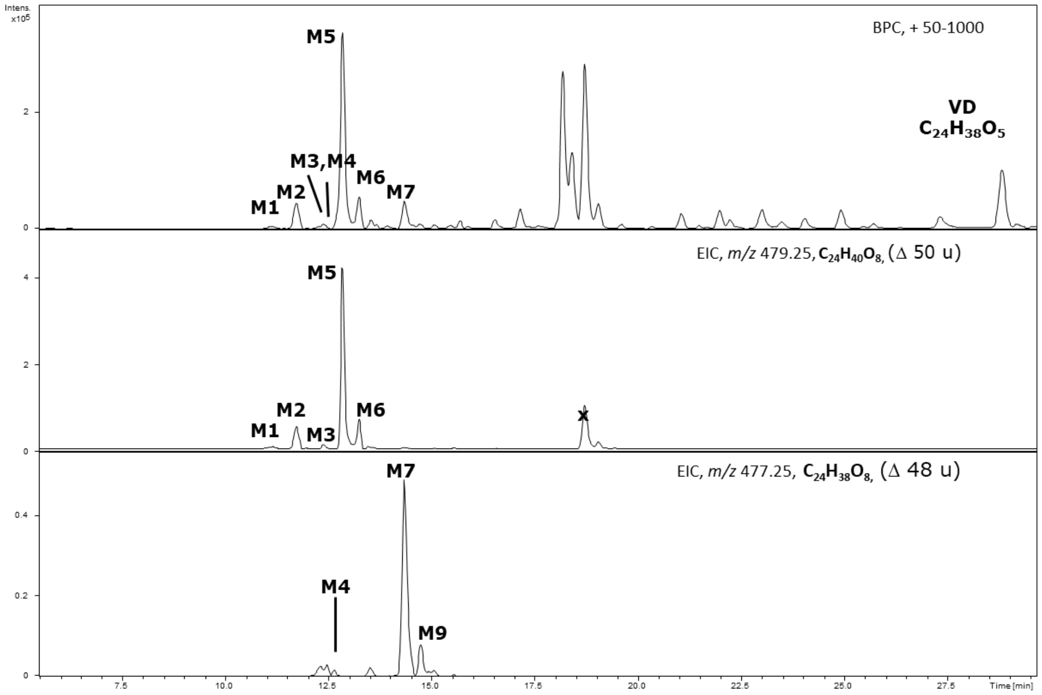

Figure S4: HPLC-DAD-QTOF-MS derived extracted ion chromatograms (EICs) for threefold oxidized metabolites generated in the HLM incubation experiment with 100 μM VD.

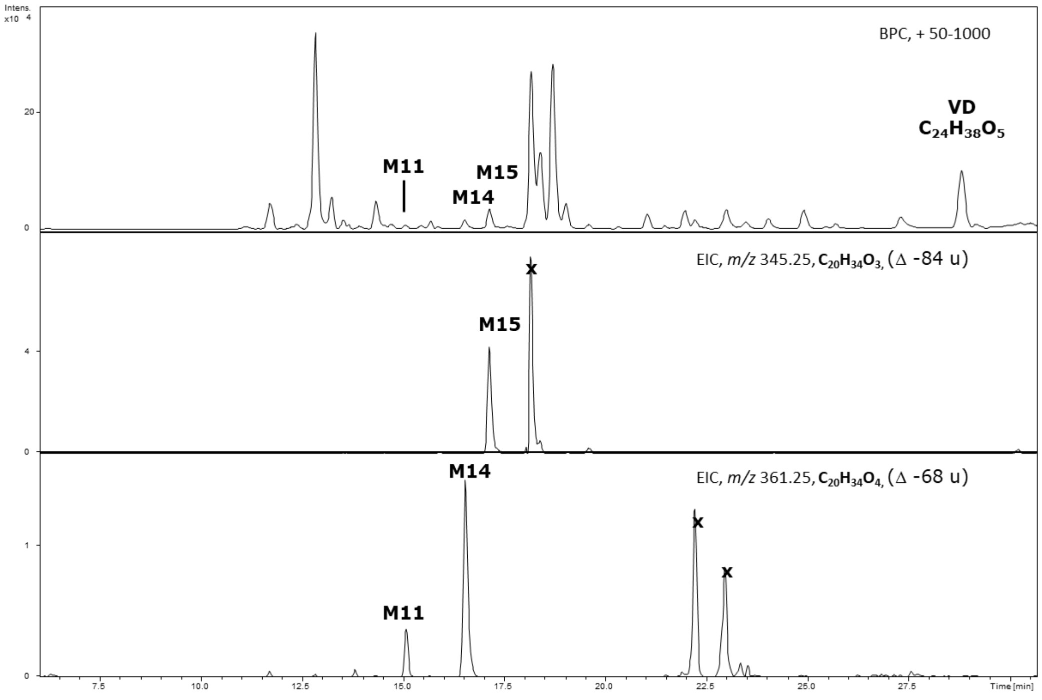

Figure S5: HPLC-DAD-QTOF-MS derived extracted ion chromatograms (EICs) for hydrolyzed metabolites generated in the HLM incubation experiment with 100 μM VD.

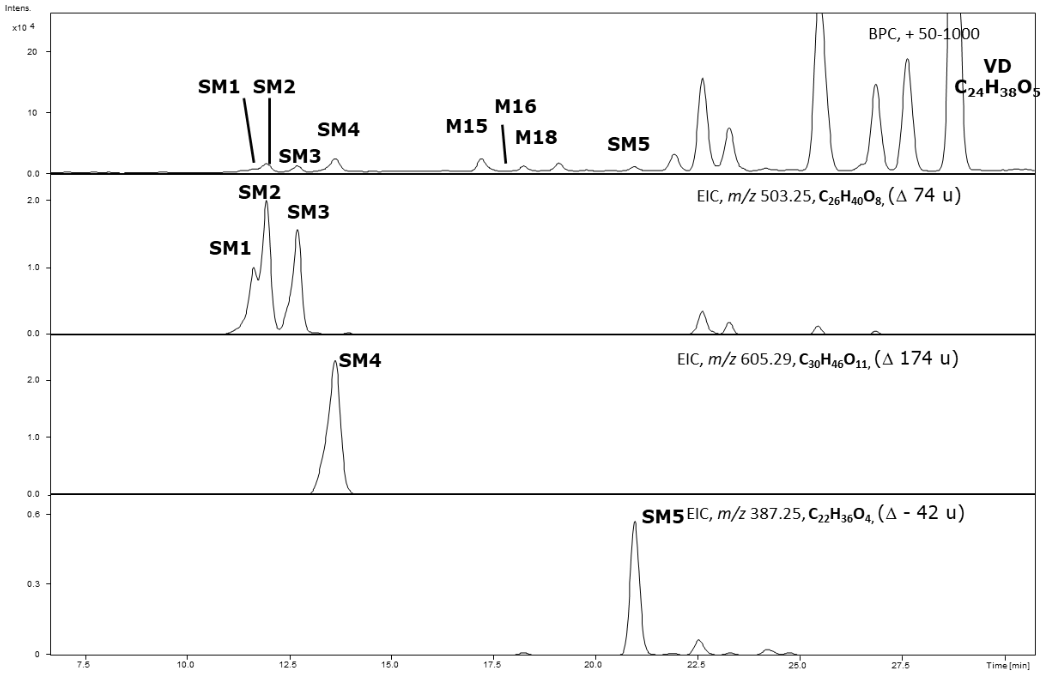

Figure S6: HPLC-DAD-QTOF-MS derived extracted ion chromatograms (EICs) for glucuroniated metabolites generated in the S9-UGT incubation experiment with 100 μM VD.

Table S1: ^{1}H (600 MHz)- and ^{13}C (150 MHz) NMR-Data of VD (CD_3CN).

Position	δ_H [ppm]	δ_H Multiplizität	*J* [Hz]	δ_C [ppm]
1	2.04<br>2.04	m<br>m	-	26.9 (CH_2)
2	1.62<br>1.62	m<br>m	-	20.5 (CH_2)
3	1.49<br>1.49	m<br>m	-	40.5 (CH_2)
4	-	-	-	35.8 (C)
5	-	-	-	133.5 (C)
6	5.57	d	3.2	67.1 (CH)
7	4.77	dd	3.2, 13.0	73.9 (CH)
8	2.04	m	-	37.8 (CH)
9	-	-	-	44.1 (C)
10	-	-	-	143.4 (C)
11	1.43<br>1.43	m<br>m	-	30.6 (CH_2)
12	1.11<br>1.43	m<br>m	-	40.5 (CH_2)
13	-	-	-	73.6 (C)
14	5.85	dd	10.7, 17.4	146.4 (CH)
15	4.99<br>5.12	dd<br>dd	1.7, 10.8<br>1.7, 17.4	112.1 (CH_2)
16	1.16	s	-	28.2 (CH_3)
17	0.92	d	7.0	11.7 (CH_3)
18	1.03	s	-	29.9 (CH_3)
19	0.88	s	-	28.7 (CH_3)
20	1.04	s	-	28.9 (CH_3)
21	-	-	-	172.1 (CO)
22	1.97	s	-	21.8 ($COCH_3$)
23	-	-	-	171.9 (CO)
24	1.91	s	-	21.4 ($COCH_3$)
OH (C13)	2.62	-	-	-

Table S2: ^{1}H (600 MHz)- and ^{13}C (150 MHz) NMR-Data of M2 (CD_3CN).

Position	δ_H [ppm]	δ_H Multiplicity	*J* [Hz]	δ_C [ppm]
1	1.85<br>2.42	m<br>dd	-<br>4.3, 16.4	36.7 (CH2)
2	3.82	m	-	65.0 (C)
3	1.38<br>1.71	t<br>ddd	12.1<br>2.1, 3.6, 12.1	48.9 (CH2)
4	-	-	-	38.3 (C)
5	-	-	-	133.5 (C)
6	5.51	d	3.1	66.4 (CH)
7	4.82	dd	3.3, 12.9	73.6 (CH)
8	2.07	dd	6.2, 13.0	37.6 (CH)
9	-	-	-	44.3 (C)
10	-	-	-	140.9 (C)
11	1.59<br>1.59	m<br>m	-	29.5 (CH2)
12	0.93<br>1.59	m<br>m	-	36.2 (CH2)
13	-	-	-	74.5 (C)
14	3.35	ddd	3.8, 5.1, 7.4	77.5 (CH)
15	3.42<br>3.60	ddd<br>ddd	5.1, 7.4, 10.9<br>3.9, 6.0, 10.8	63.8 (CH2)
16	1.06	s	-	23.2 (CH3)
17	0.96	d	7.1	11.7 (CH3)
18	1.06	s	-	30.4 (CH_3)
19	0.90	s	-	28.7 (CH_3)
20	1.08	s	-	28.5 (CH_3)
21	-	-	-	172.1 (CO)
22	1.98	s	-	22.1 ($COCH_3$)
23	-	-	-	171.9 (CO)
Ac-23	1.91	s	-	21.6 ($COCH_3$)
OH (C2)	2.79	-	-	-
OH (C13)	2.78	-	-	-
OH (C14)	3.05	d	5.1	-
OH (C15)	2.84	dd	5.1	-

Table S3: ^{1}H (600 MHz)- and ^{13}C (150 MHz) NMR-Data of M5 (CD_3CN).

Position	δ_H [ppm]	δ_H Multiplicity	*J* [Hz]	δ_C [ppm]
1	2.07<br>2.29	m<br>ddd	-<br>5.9, 9.2, 17.4	(CH2)
2	1.64<br>1.81	m<br>m	-	(CH2)
3	3.45	m	-	75.5 (C)
4	-	-	-	40.6 (C)
5	-	-	-	131.8 (C)
6	5.57	d	3.3	66.8 (CH)
7	4.84	dd	3.3, 12.9	74.3 (CH)
8	2.07	m	-	38.2 (CH)
9	-	-	-	43.5 (C)
10	-	-	-	142.3 (C)
11	1.51<br>1.51	m<br>m	-	29.9 (CH2)
12	0.95<br>1.51	m<br>m	-	38.3 (CH2)
13	-	-	-	75.2 (C)
14	3.35	ddd	3.8, 5.1, 7.4	76.7 (CH)
15	3.41<br>3.59	ddd<br>ddd	5.1, 7.4, 10.8<br>3.8, 6.0, 10.8	(CH2)
16	1.04	s	-	23.1 (CH3)
17	0.95	d	7.0	11.3 (CH3)
18	1.02	s	-	28.9 (CH3)
19	0.89	s	-	22.4 (CH3)
20	1.09	s	-	28.6 (CH3)
21	-	-	-	171.6 (CO)
22	1.98	s	-	21.0 (COCH3)
23	-	-	-	171.5 (CO)
24	1.91	s	-	20.9 (COCH3)
OH (C3)	2.68	-	-	-
OH (C13)	2.76	-	-	-
OH (C14)	3.04	d	5.1	-
OH (C15)	2.82	dd	5.1	-

Table S4: ^{1}H (600 MHz)- and ^{13}C (150 MHz) NMR-Data of M15 (CD_3CN).

Position	δ_H [ppm]	δ_H Multiplicity	*J* [Hz]	δ_C [ppm]
1	1.98<br>1.98	m<br>m	-	27.1 (CH_2)
2	1.60<br>1.60	m<br>m	-	21.1 (CH_2)
3	1.48<br>1.48	m<br>m	-	41.2 (CH_2)
4	-	-	-	35.6 (C)
5	-	-	-	137.9 (C)
6	3.96	m	-	67.9 (CH)
7	3.34	ddd	3.6, 8.4, 12.2	72.9 (CH)
8	1.69	m	-	40.6 (CH)
9	-	-	-	43.5 (C)
10	-	-	-	139.7 (C)
11	1.34<br>1.40	m<br>m	-	30.9 (CH_2)
12	1.07<br>1.37	m	-	40.7 (CH_2)
13	-	-	-	73.8 (C)
14	5.85	dd	10.7, 17.4	147.2 (CH)
15	4.97<br>5.11	dd<br>dd	1.7, 10.7<br>1.7, 17.4	112.1 (CH_2)
16	1.14	s	-	28.6 (CH_3)
17	0.99	d	6.9	12.5 (CH_3)
18	1.13	s	-	30.4 (CH_3)
19	1.04	s	-	30.4 (CH_3)
20	0.97	s	-	29.2 (CH_3)
OH (C6)	2.46	-	-	-
OH (C7)	2.54	-	-	-

Table S5: ^{1}H (600 MHz)- and ^{13}C (150 MHz) NMR-Data of M16 (CD_3CN).

Position	δ_H [ppm]	δ_H Multiplicity	*J* [Hz]	δ_C [ppm]
1	1.82<br>2.40	dd<br>dd	6.0, 16.4<br>4.4, 16.4	36.9 (CH_2)
2	3.83	m	-	65.3 (C)
3	1.37<br>1.71	t<br>ddd	12.1<br>3.4, 12.3	49.3 (CH_2)
4	-	-	-	38.4 (C)
5	-	-	-	133.5 (C)
6	5.51	d	3.20	66.7 (CH)
7	4.76	dd	3.2, 13.0	74.0 (CH)
8	2.06	dd	6.2, 13.0	37.8 (CH)
9	-	-	-	44.0 (C)
10	-	-	-	141.3 (C)
11	1.45<br>1.45	m<br>m	-	30.3 (CH_2)
12	1.07<br>1.45	m<br>m	-	40.5 (CH_2)
13	-	-	-	73.8 (C)
14	5.85	dd	10.7, 17.4	146.8 (CH)
15	5.00<br>5.13	dd<br>dd	1.7, 10.7<br>1.7, 17.4	112.3 (CH_2)
16	1.16	s	-	28.4 (CH_3)
17	0.92	d	7.0	11.9 (CH_3)
18	1.06	s	-	30.6 (CH_3)
19	0.92	s	-	28.9 (CH_3)
20	1.05	s	-	28.7 (CH_3)
21	-	-	-	172.2 (CO)
22	1.97	s	-	21.9 ($COCH_3$)
23	-	-	-	171.9 (CO)
24	1.91	s	-	21.5 ($COCH_3$)
OH (C2)	2.77	dd	1.2, 4.7	-
OH (C13)	2.62	-	-	-

Table S6: ^{1}H (600 MHz)- and ^{13}C (150 MHz) NMR-Data of M17 (CD_3CN).

Position	δ_H [ppm]	δ_H Multiplicity	*J* [Hz]	δ_C [ppm]
1	1.99<br>2.27	m<br>ddd	-<br>5.9, 9.2, 17.4	22.8 (CH_2)
2	1.64<br>1.81	m<br>m	-	27.5 (CH_2)
3	3.44	m	-	75.1 (C)
4	-	-	-	40.4 (C)
5	-	-	-	132.7 (C)
6	5.57	d	3.1	67.3 (CH)
7	4.78	dd	3.2, 12.9	74.0 (CH)
8	2.05	ddd	7.1, 7.1, 13.0	37.9 (CH)
9	-	-	-	44.1 (C)
10	-	-	-	143.0 (C)
11	1.44<br>1.44	m<br>m	-	30.6 (CH_2)
12	1.11<br>1.44	m<br>m	-	40.9 (CH_2)
13	-	-	-	73.5 (C)
14	5.85	dd	10.8, 17.3	146.8 (CH)
15	4.98<br>5.12	dd<br>dd	1.5, 10.8<br>1.5, 17.3	111.9 (CH_2)
16	1.16	s	-	28.5 (CH_3)
17	0.92	d	7.1	11.9 (CH_3)
18	1.03	s	-	29.1 (CH_3)
19	0.88	s	-	22.9 (CH_3)
20	1.06	s	-	29.1 (CH_3)
21	-	-	-	171.9 (CO)
22	1.98	s	-	21.9 ($COCH_3$)
23	-	-	-	171.9 (CO)t
24	1.91	s	-	21.6 ($COCH_3$)
OH (C3)	2.67	-	-	-
OH (C13)	2.62	-	-	-

Table S7: ^1H (600 MHz)- and ^{13}C (150 MHz) NMR-Data of M19 (CD_3CN).

Position	δ_H [ppm]	δ_H Multiplicity	*J* [Hz]	δ_C [ppm]
1	2.04<br>2.10	m<br>m	-	27.0 (CH2)
2	1.62<br>1.62	m<br>m	-	20.5 (CH2)
3	1.49<br>1.49	m<br>m	-	40.5 (CH2)
4	-	-	-	35.6 (C)
5	-	-	-	133.7 (C)
6	5.57	d	3.2	67.1 (CH)
7	4.81	dd	3.3, 12.7	74.4 (CH)
8	2.05	m	-	37.8 (CH)
9	-	-	-	44.3 (C)
10	-	-	-	143.6 (C)
11	1.49<br>1.49	m<br>m	-	30.2 (CH2)
12	1.05<br>1.49	m<br>m	-	41.1 (CH2)
13	-	-	-	75.0 (C)
14	3.35	ddd	3.6, 5.5, 7.4	76.8 (CH)
15	3.46<br>3.56	ddd<br>ddd	4.7, 7.4, 11.2<br>3.4, 6.4, 10.8	64.5 (CH2)
16	1.03	s	-	23.4 (CH3)
17	0.94	d	7.1	12.0 (CH3)
18	1.04	s	-	30.1 (CH3)
19	0.88	s	-	29.1 (CH3)
20	1.06	s	-	29.3 (CH3)
21	-	-	-	171.9 (CO)
22	1.98	s	-	21.6 (COCH3)
23	-	-	-	171.8 (CO)
24	1.91	s	-	21.6 (COCH3)
OH (C14)	3.02	-	-	-

Table S8: ^{1}H (600 MHz)- and ^{13}C (150 MHz) NMR-Data of M20 (CD_3CN).

Position	δ_H [ppm]	δ_H Multiplicity	*J* [Hz]	δ_C [ppm]
1	n.d.[a]	-	-	n.d.[a]
2	n.d.[a]	-	-	n.d.[a]
3	3.42	m	-	75.7 (CH)[b]
4	-	-	-	40.6 (C)
5	-	-	-	133.7 (C)
6	5.53	d	3.3	n.d.[a]
7	4.78	dd	3.3, 13.0	75.8 (CH)[b]
8	2.05	m	-	37.2 (CH)
9	-	-	-	43.2 (C)
10	-	-	-	141.8 (C)
11	-[b]	-[b]	-	30.6 (CH2)
12	-[b]	-[b]	-	39.8 (CH2)
13	-	-	-	73.0 (C)
14	5.84	ddd	10.8, 17.4	146.1 (CH)
15	4.98<br>5.12	dd<br>dd	1.6, 10.7<br>1.6, 17.4	n.d.[a]
16	1.16	s	-	28.2 (CH_3)
17	0.92	d	7.1	11.6 (CH_3)
18	0.95	s	-	22.8 (CH_3)
19	0.91	s	-	24.4 (CH_3)
20	1.04	s	-	28.8 (CH_3)
21	-	-	-	171.3 (CO)
22	1.98	s	-	21.5 ($COCH_3$)
23	-	-	-	171.7 (CO)
24	1.91	s	-	21.2 ($COCH_3$)

[a] not detected, [b] interchangeable

Table S9: ^{1}H (600 MHz)- and ^{13}C (150 MHz) NMR-Data of M22 (CD_3CN).

Position	δH [ppm]	δH Multiplicity	*J* [Hz]	δC [ppm]
1	n.d.[a]	-	-	n.d.[a]
2	n.d.[a]	-	-	n.d.[a]
3	-	-	-	215.0 (C)
4	-	-	-	48.7 (C)
5	-	-	-	132.7 (C)
6	5.58	d	3.2	66.9 (CH)
7	4.85	dd	3.2, 13.0	74.2 (CH)
8	2.01	m	-	38.4 (CH)
9	-	-	-	44.6 (C)
10	-	-	-	145.6 (C)
11	1.48<br>1.48	m<br>m	-	30.4 (CH_2)
12	1.13<br>1.48	m<br>m	-	40.4 (CH_2)
13	-	-	-	73.6 (C)
14	5.85	dd	10.8, 17.4	147.7 (CH)
15	4.99<br>5.14	dd<br>dd	1.6, 10.7<br>1.6, 17.4	112.0 (CH_2)
16	1.16	s	-	29.3 (CH_3)
17	0.95	d	7.0	11.5 (CH_3)
18/19[b]	1.16	s	-	25.6 (CH_3)
18/19 [b]	1.04	s	-	24.6 (CH_3)
20	1.13	s	-	28.8 (CH_3)
21	-	-	-	171.9 (CO)
22	1.99	s	-	21.8 ($COCH_3$)
23	-		-	171.0 (CO)
24	1.93	s	-	21.3 ($COCH_3$)

[a] not detected
